# A novel secondary structure based on fused five-membered rings motif

**DOI:** 10.1038/srep31483

**Published:** 2016-08-11

**Authors:** Jesmita Dhar, Raghuvansh Kishore, Pinak Chakrabarti

**Affiliations:** 1 Bioinformatics Centre, Bose Institute, P1/12 CIT Scheme VIIM, Kolkata 700054, India; 2 Protein Science & Engineering Division, CSIR-Institute of Microbial Technology, Sector 39-A, Chandigarh 160 036, India; 3 Department of Biochemistry, Bose Institute, P1/12 CIT Scheme VIIM, Kolkata 700054, India

## Abstract

An analysis of protein structures indicates the existence of a novel, fused five-membered rings motif, comprising of two residues (i and i + 1), stabilized by interresidue N_i+1_–H∙∙∙N_i_ and intraresidue N_i+1_–H∙∙∙O=C_i+1_ hydrogen bonds. Fused-rings geometry is the common thread running through many commonly occurring motifs, such as β-turn, β-bulge, Asx-turn, Ser/Thr-turn, Schellman motif, and points to its structural robustness. A location close to the beginning of a β-strand is rather common for the motif. Devoid of side chain, Gly seems to be a key player in this motif, occurring at i, for which the backbone torsion angles cluster at ~(−90°, −10°) and (70°, 20°). The fused-rings structures, distant from each other in sequence, can hydrogen bond with each other, and the two segments aligned to each other in a parallel fashion, give rise to a novel secondary structure, *topi*, which is quite common in proteins, distinct from two major secondary structures, α-helix and β-sheet. Majority of the peptide segments making *topi* are identified as aggregation-prone and the residues tend to be conserved among homologous proteins.

Information from protein and small molecule structures has been complimentary to each other, refining our knowledge on diverse non-covalent interaction. For example, aromatic-aromatic interactions seen in proteins^1^ have been rationalized from our observations in small molecule structures^2^. Similarly, turn conformations involving *cis* peptide bonds in protein structures^3^ have been shown to exist even in isolated peptides bereft of the protein scaffold^4^,^5^. On the other hand, C–H∙∙∙O interaction seen in peptide structures^6^ has been found to be of common occurrence in proteins leading to the identification of ω-turn, a new type of β-turn mimic^7^. Both conventional and non-conventional hydrogen bonds, such as C–H∙∙∙π interactions, seen in small molecule crystals are also important for the structure and function of protein molecules^8^,^9^.

Short-range interactions lead to motifs, which are structurally stable and easily identifiable in both protein and peptide structures. One such motif involves two consecutive residues where the N–H group of the second points towards the lone-pair electrons on the main-chain N of the first residue^10^. Usually, the N–H group is also associated with another conventional hydrogen bond (Fig. 1a). Yet another two-residue motif is the basic unit of 2.0_5_-helix^11^, which has the backbone in the fully-extended conformation (ϕ, ψ both ≈ 180°). This ‘planar sheet’ structure was originally proposed by Pauling and Corey and shown to be less stable than pleated β-sheet structures for all amino acids except Gly^12^. This motif is defined by pentagonal intramolecularly hydrogen-bonded structure, the so-called C_5_ conformation (Fig. 1b), which has been observed in model compounds and proteins in Gly-containing stretche^13^,^14^. Although the N–H∙∙∙O angle in the motif deviates considerably from linearity (lying in the range ~90° to 110°) the evidence for the occurrence of the intra-residue hydrogen bond has come from FT-IR and NMR experimental data acquired on a number of peptides containing non-proteinogenic, C^α,α^-disubsituted Gly residues, and also from the fact that in many of the crystal structures the internal N–H donors and C=O acceptors do not participate in any competing intermolecular hydrogen bonds^15^.

The two motifs discussed above were found to occur together in the peptide, Boc-Leu-Thr-NH_2_ (where Boc = *t*-butoxycarbonyl group)^16^ (also unpublished data) (Fig. 1c providing an equivalent fused five-membered rings motif seen in protein structures). In the fused-rings motif, while the first residue prefers a semi-folded conformation *i.e.*, ϕ ~ −120° and ψ ~ 0° being in the folded “bridge” region, the second residue adopts a significantly extended conformation *i.*e., ϕ ~ − 140° and ψ ~ 160°. The structure, stabilized by N_i+1_–H∙∙∙N_i_ and N_i+1_–H∙∙∙O=C_i+1_ interactions, has a rather planar topology, which is predominantly retained in solution, as evidenced from ^1^H NMR and FT-IR spectroscopic data.

In this paper we address two questions. First, the two residues that were earlier considered^10^ for exhibiting N_i+1_–H∙∙∙N_i_ interaction, contained Pro at the *i*^th^ position (Fig. 1a). We wanted to know the prevalence of the interaction in protein structures and the propensity of residues to be part of this. Secondly, if the fused-rings structure, as characterized in short peptide, is found in proteins as well. Our analysis establishes that the fused-rings motif is very common in protein structure, and in combination with additional hydrogen bond, this is part of various known structural elements. More importantly, we have even identified the motif to generate unique secondary structure. The results have implications for protein folding problem as it is seen that the two fused-rings motifs, distant in sequence along the protein, can recognize and interact with each other giving rise to structure with distinct topology.

## Results

### N–H∙∙∙N(p_z_) interaction involving consecutive peptide groups

**Figure 1:**
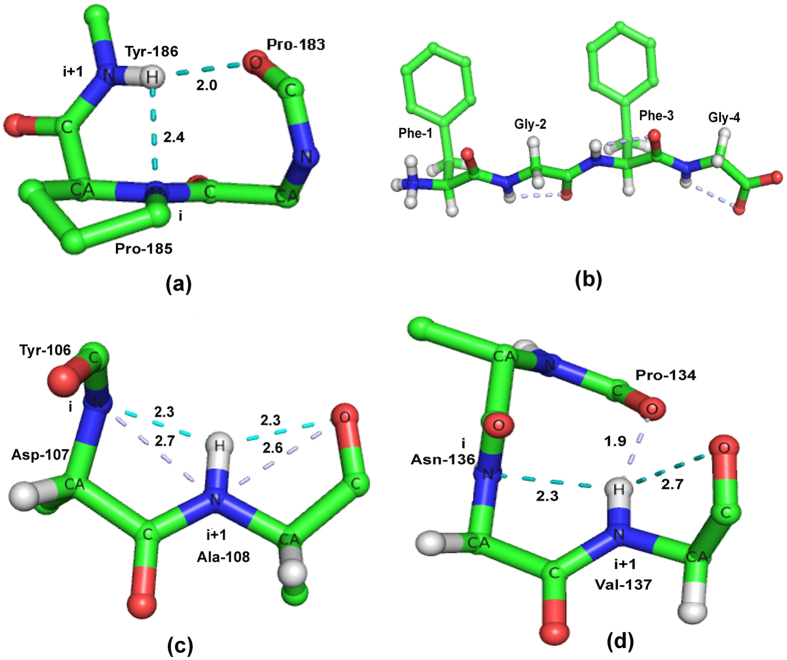
Some hydrogen-bonded motifs and the structures where they occur. (**a**) An N–H group involved in N–H···N(p_z_) interaction with the preceding peptide group, and also participating in another hydrogen bond (taken from the PDB file, 1CKA)^10^. (**b**) Hydrogen-bonded C_5_ conformation occurring in a peptide structure with the sequence Phe-Gly-Phe-Gly^50^. (**c**) Fused five-membered rings motif in 1B25. (**d**) The fused-rings motif, with the N–H forming an additional hydrogen bond in 1A68. The relevant hydrogen bond distances (Å) are indicated as dashed lines.

The distribution of θ, the angle made by the peptide hydrogen atom with the perpendicular direction to the peptide N atom of the previous residue, is shown in Fig. 1 in Supplementary. Comparison with the expected distribution, represented by a sine function, suggests a very stereospecific and stabilizing interaction occurring between the two neighboring peptide groups when θ is <30°. The typical N–H∙∙∙N interactions, for example, those connecting the DNA bases, occur along the planes of the interacting moieties^17^. Here, as the hydrogen bond involves the p_z_ orbital of the acceptor it is designated as N–H∙∙∙N(p_z_) interactions and appears to be very common in protein structures (Table 1). Sporadic cases of such interactions have been reported before^18^, and also implicated when the side chain –NH_3_^+^ of Lys was found sitting on the face of the ring N atoms of His residue^19^. As has been observed with the limited number of cases^10^, the N–H group in 64% of those having N–H∙∙∙N(p_z_) interactions can participate in additional hydrogen bonding with other protein acceptors (Fig. 1a) (1% have two additional interactions).

### Occurrence of fused C_5_ rings motifs

A total 41440 cases, 54% of those exhibiting N–H∙∙∙N(p_z_) hydrogen bonds, are part of fused five-membered (C_5_) rings motifs (Fig. 1c), which corresponds to 4.1(±2.2) occurrences per 100 residues (an average of ~10 motifs per protein chain). The average nonbonded distances are N_i+1_–H···N_i_, 2.4(±0.12) Å and N_i+1_–H···O_i+1_, 2.59(±0.25) Å and the average angles are ∠N_i+1_–H···N_i_, 98.2(±4)° and ∠N_i+1_–H···O_i+1_, 90(±12)°. As can be seen from Table 1, 53% of the N–H group in fused rings participate in additional hydrogen bonds, which could be short (within four residues, 15125 cases, (Fig. 1d)), or long (beyond 4 residues) range. In the fused-rings motif, the N_i+1_–H donor (D) interacting with two acceptors (N_i_ and O_i+1_) (A) is an example of 1D-2A type of hydrogen bond. The same donor interacting with another acceptor would lead to the type 1D-3A.

### Conformational features of residues involved in fused-rings motif

Consideration of the backbone angles of the two residues indicated that the residue at position i can have two sets of values related by centre of inversion, whereas the one at i + 1 has nearly identical average values (though the distribution displays a rather wide spread) (Fig. 2 in Supplementary). For the two clusters (containing 69 and 31% of data points) the sets of ϕ, ψ angles are ϕ_i_ = −88(±18)°, ψ_i_ = −11(±22)° and ϕ_i+1_ = −126(±32)°, ψ_i+1_ = 136(±40)°; and ϕ_i_ = 73(±15)°, ψ_i_ = 20(±20)° and ϕ_i+1_ = −116(±22)°, ψ_i+1_ = 141(±34)°, respectively. In the second cluster (with positive ϕ_i_) the percentage of Gly, Asp and Asn are 59, 6 and 12, respectively. The two sets of ϕ, ψ angles correspond to the flipping of the peptide group (between i-1 and i residues), such that N_i+1_–H interacts with the lone pair of electrons on N_i_ atom on either side of the plane. Interestingly, ϕ_i_, ψ_i_ values follow a rather linear distribution, an increase in ϕ leads to a concomitant decrease in ψ of residue i, such that N–H proton of residue i + 1 can have maximum interaction with the N(p_z_) orbital of the preceding residue, again showing the importance of this interaction to the stability of protein three-dimensional structures.

### Residue propensity

**Figure 2:**
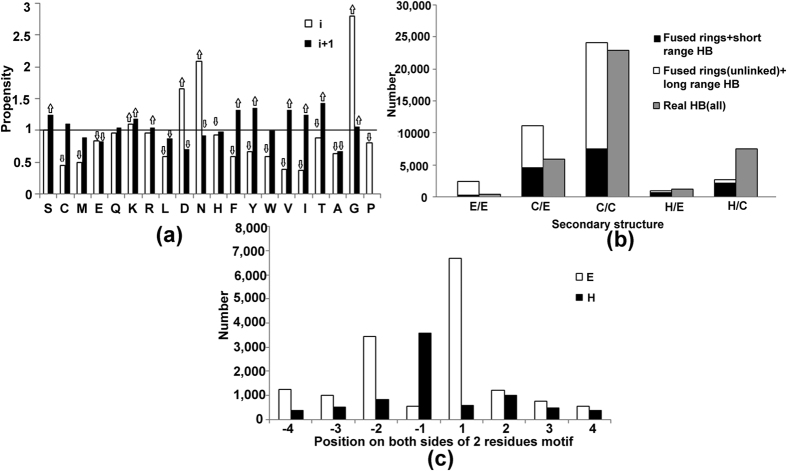
Propensities and secondary structural features. (**a**) Propensities of residues to occur at i and i + 1 positions of the fused-rings motifs. (Very similar values were obtained when calculations were done using those structures that contain the motif as well as additional short-range hydrogen bond). Up arrow (⇑) indicates over-representation and down arrow (⇓) indicates under-representation. (**b**) Secondary structural preferences for residues (at i and i + 1 positions) across the fused-rings motif, and its subgroup having additional short/long-range hydrogen bond outside the motif. Secondary structures are indicated by H, E and C (explained in Methods); H/C indicates H and C to be the secondary structures of the two residues. Only the combinations that are observed are indicated. (**c**) The first occurrence of secondary structures (H or E) on either side of the fused-rings motifs (participating in additional short range hydrogen bond), up to 4 residues, before position i or after i + 1.

The propensities of a residue to occur at i and i + 1 positions were calculated as the ratio of the percentage of occurrence of that particular residue at either of these positions to its percentage in the whole database. The significance of the result is indicated by the *z* values^20^, which are presented in Table 1 (in supplementary). Gly, Asn and Asp, with high propensities are over-represented at position i (Fig. 2a). The preference for these residues, especially non-chiral Gly, is not surprising, considering the fact that a positive value of ϕ is also favored at this position. Although an earlier experimental work^10^ involved cases with Pro at position i, this residue is one of the least favored. At the (i + 1)th position, hydroxyl-containing (Ser, Thr and Tyr), and nonpolar residues, such as Phe, Val and Ile are over-represented. Interestingly, residues (Asp, Asn, Phe, Tyr, Val, Ile and Thr), if over-represented in one position is under-represented in the other. Ala, Leu and Glu are under-represented at both the positions.

### Secondary structures of residues making up the fused-rings motif and those flanking it

We also identified the secondary structural features for both the residues at i and i + 1 of fused rings and observed that the maximum preference is for C/C (58%) followed by C/E (27%) (Fig. 2b); the preference does not change much if we consider those cases which have additional hydrogen bond interactions outside the motif.

We found out the nearest regular secondary structure (H or E) that occurs within four residues on either side (4 residues, upstream of i or downstream of i + 1); we restricted ourselves to only those cases where the NH group of the motif also participates in additional short range hydrogen bond (dealt in the next section). Figure 2(c) indicates that β-strand is the most common secondary structure to be found in the neighborhood of the motif, in particular immediately following it. Therefore, the motif appears to be a good initiator of β-strand, which is facilitated by the ϕ, ψ angles at position i + 1 being close to those expected for residues in β-sheet and also by the preference of typical β-sheet residues (such as those with branched side-chain) at this position. The motif can also link two β-strands, which is apparent considering the two major classes of the occurrence of secondary structures involving the motif (Fig. 2b) separately. When the fused rings have the structural combination C/C (7538 cases), β-strand precedes and follows these in 25 and 50% cases, respectively (Fig. 3a in Supplementary). Even when the combination is C/E (which implies that the motif is the starting point of a β-strand), a strand is likely to precede it at −2 position (67% of 4545 cases) (Fig. 3b in Supplementary).

### Local structural features involving fused-rings motif with N–H having additional hydrogen bond (1D-3A)

Considering the residue at position i + 1 of the fused rings as the pivotal residue we found out the sequence difference from the residue to which it can form additional short range hydrogen bond. The data in Table 2 indicate that the sequence difference is overwhelmingly positive, such that the hydrogen bond is with a residue that precedes it. The maximum number (71% of 15125) is with D = 3, followed by 2 (14%) and 4 (11%). Except for D = 2, the interaction is essentially with the main chain. When D = 0 (33 (0.2%) cases) the interaction is with the side-chain O atom of the same residue.

**Figure 3:**
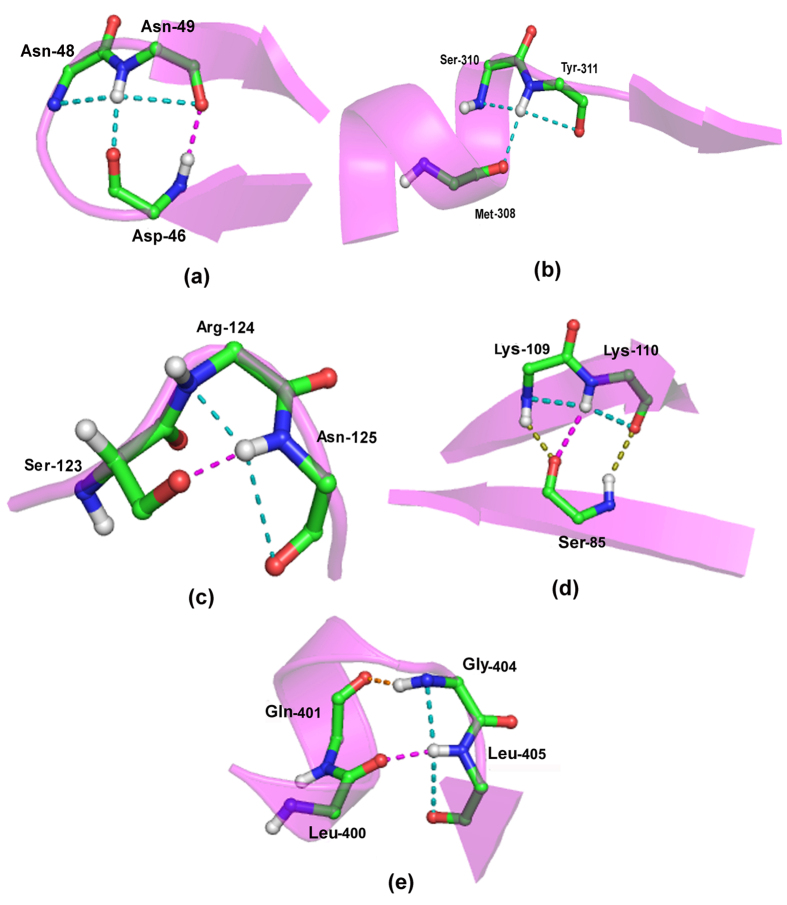
Some examples of fused-rings motifs having additional short-range (D = 3 in (**a**,**b**), 2 in (**c**) and long-range (**d**,**e**)) interactions. (**a**) The motif is part of type II’ β-turn, located between two β-strands (PDB, 1B2P); hydrogen bonds within the motif are in cyan, and the first interaction between the two strands is shown as pink broken line. (**b**) The motif is constituent of type I β-turn, located between a β-strand and a helix (PDB, 1B6A). (**c**) The motif is part of Ser/Thr turn (PDB, 1CB8). (**d**) The motif located in a β-strand is interacting with the main-chain atoms of an adjacent antiparallel strand, forming β-bulge structure (PDB, 1OEW). (**e**) The motif along with the two hydrogen bonds at helix C-terminus that define the Schellman motif, which is followed by a strand in the PDB file, 2W1Z.

The involvement of the main-chain atom for hydrogen bonding and the conformational feature (ϕ, ψ values of (−88°, −11°) or (73°, 20°)) of the pivotal residue made us analyze if this could simultaneously be the second of the two central residues in β-turns^21^. Though not commented upon earlier, this can indeed be the case, and two examples of occurrence in types II’ and I β-turns, when D = 3, are shown in Fig. 3a,b. The identification of the flanking secondary structures (Table 2 in Supplementary) indicates that types I’ and II’ are usually found in β-hairpins, as noted earlier^21^, but occurring between a helix and β-strand the fused-rings motif is usually of type I (Fig. 3b). D = 2 is the only category that has a large involvement of the side-chain atoms. Based on the residue preference at different positions in this segment (Fig. 4 in Supplementary) one can see that the motif is part of Ser/Thr turn (64%) or Asx-turn (30%) (Fig. 3c)^22^. Interestingly, in the 3-residue peptide segment, the second position is predominantly occupied by Gly, as can be expected at the *i*th position of the fused-rings motif (Fig. 2a). When we considered the 6802 cases involved in long range interactions we again found a number of them to be part of known structural motifs, such as β-bulge (18%) and Schellman motif (34%). A β-bulge occurs between two antiparallel strands such that the extreme NH and CO groups of two residues in one strand engage CO and NH of one residue in the opposing strand^23^. As can be seen from Fig. 3d, the hydrogen bond criteria of fused ring and β-bulge can be satisfied simultaneously. Schellman motif is the most prominent capping feature at the C-termini of helices^24^. It consists of six residues (m to m + 5), the first three are part of the helix and the next two belong to turn; typically there are two hydrogen bonds m to (m + 5), and (m + 1) to (m + 4), and the residue at (m + 4) normally has + ve ϕ value with a preference for Gly^25^. It is evident from Fig. 3e that (m + 4) and (m + 5) positions of the Schellman motif may also constitute the fused-rings motif. Because of the higher tendency of the fused-rings motif to be followed by strand (Fig. 2c), 44% of Schellman motifs in our study lead to a β-strand.

### Structures with linked fused-rings motif

**Figure 4:**
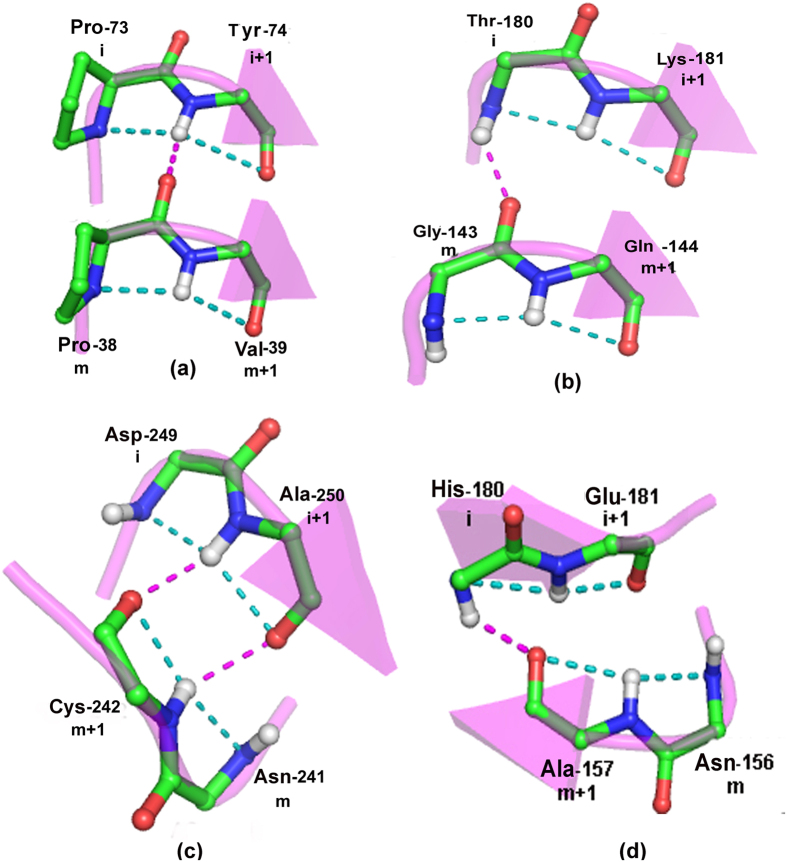
Some illustrative examples of linked fused-rings motifs exhibiting long range interaction (represented in pink broken line). The different categories of interactions, depending on the positions of the donor and acceptor, are (**a**) (i + 1) → m (PDB, 1BYI), (**b**) i → m (PDB, 1CVR), (**c**) (i + 1) → (m + 1) (PDB, 2BZV) and (**d**) i → (m + 1) (PDB, 3P2C).

We made another interesting observation while addressing the question if two fused rings can be found hydrogen bonded to each other in protein structures. The hydrogen bond can involve not only the N–H group of the fused rings, but also the carbonyl groups of the motif. Depending on which group is involved the interaction can be designated based on the two residue labels (i, i + 1, and m, m + 1) of the two fused rings. These are (i + 1) → m, i → m, (i + 1) → (m + 1) and i → (m + 1), Fig. 4 providing the illustrative examples. Their number (170, 337, 24 and 155, respectively) indicates that the second group predominates, though the first and the fourth also make substantial contribution. The individual cases are given in Table 3 in Supplementary, which also indicates if the two peptide segments containing the fused rings are parallel or antiparallel to each other, although in some cases, when the two stretches are not aligned to each other, their occurrence may not be easily ascertained. The first group (Fig. 4a) is the most regular where the two motifs are aligned, back-to-back. This is also true for the second group when the segments are parallel (though the antiparallel orientation has the higher occurrence). For the last two groups the motifs face each other, with varying angle between them.

**Figure 5:**
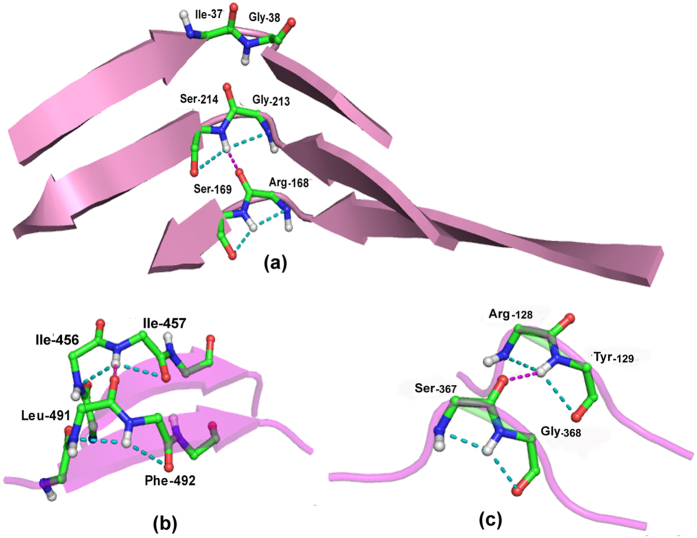
Some examples of the simultaneous occurrence of two fused-rings motifs, connected by a long range hydrogen bond, (i + 1) → m. Individual motifs occurs between two β-strands in (**a**) (PDB, 1B5E), in the same strand in (**b**) (PDB, 1OGO), and in loops in (**c**) (PDB, 2CHO).

**Figure 6:**
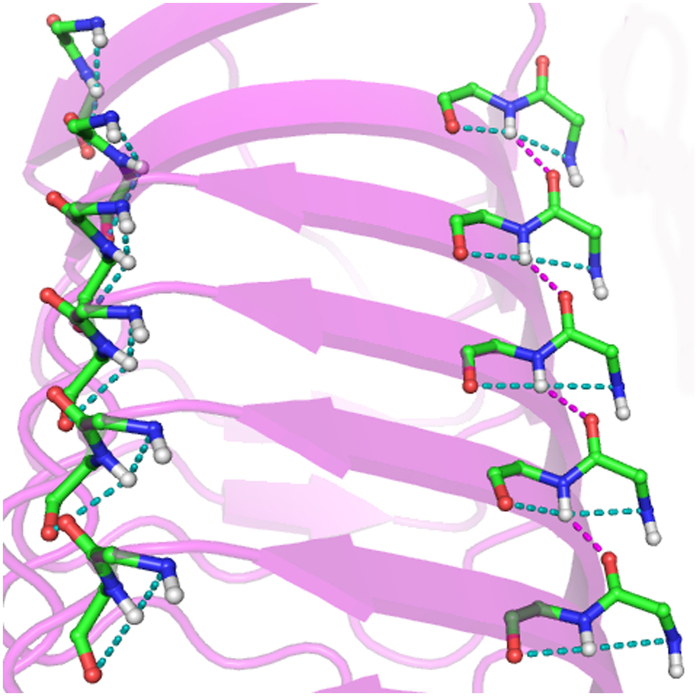
The occurrence of a series of both linked ((i + 1) → m, pink broken line) (right) and unlinked (left) fused-rings motifs in the *Efs*Qnr protein (PDB, 2W7Z).

Figure 5 provide examples of (i + 1) → m categories. It can be seen that the motifs can occur in adjacent strands (usually parallel), or can provide a short spacer between strands, or even occur in loops. Overall, the coming together of distant regions of the polypeptide chain with fused-rings motif gives rise to a unique shape, similar to that of a hat, which in most Indian languages is called *topi. Topi* is thus a new secondary structure. In fact all the individual motifs have this shape, but only when they come together and are aligned the shape gets accentuated (Fig. 4a,b,d). The secondary structure is usually made up of two fused-rings motif. Figure 5a, however, provides an interesting example where a third segment, devoid of the motif, has been added (in an antiparallel fashion), but maintaining the overall shape of the structure. The robustness of the structure becomes apparent if we look at the superfamily of pentapeptide-repeat proteins, having right-handed quadrilateral parallel β-helix fold^26^ (Fig. 6), where a series of linked fused-rings are found on parallel β-strands^27^. Noteworthy, occurring at strand ends where the loops make a sharp bend there is a series of the motifs which are not linked.

*Topi* structure has been identified by considering hydrogen bonding between any groups in the fused-rings motif. Such an approach also led to the identification of linked motifs which are very close along the sequence (Fig. 5 in Supplementary). It is obvious that such occurrence (72 pairs, with the number of intervening residues ≤4) brings order to the otherwise intractable irregular loop regions. Another example worth mentioning is the occurrence of two linked fused rings, individually forming β-bulges in the adjacent antiparallel strands (Fig. 6 in Supplementary). While a single motif is the norm (Fig. 3d), the occurrence of the motifs in both the strands, though rare (only two pairs have been observed) may be useful for accommodating single-residue insertions in the neighbouring strands without disrupting the β-sheet^23^ and also for providing strength to the bending induced in the sheet.

### Aggregation propensity and residue conservation

We found out if the sequences involved in *topi* motif have an inherent tendency to aggregate by noting if they belong to segments of at least five consecutive residues populating the β aggregated conformation, as indicated by TANGO^28^. We observed that in the 129 cases (results could not obtained for 41 motifs belonging to proteins with very long chain) of type (a) motif (Fig. 4 and Table 3 in Supplementary), both the constituent segments are susceptible to aggregation in 25% cases, one in 40%, and none in the remaining 35% cases (Table 4 in Supplementary). To take a specific example (Fig. 5a), the segment (Gly213-Ser214) in the middle has been found to have positive propensity for aggregation, which, however, is not the case with the one below (Arg168-Ser169).

We calculated the degree of conservation at i and i + 1 positions in the 340 segments (of 170 *topi* motifs considered above) and got values of 61 and 73%, respectively. The second position seems to be slightly more conserved. A possible reason could be the involvement of this residue in a greater number of hydrogen bonds (Fig. 5)–as the bonds involve the main-chain atoms the residue specific effect could only be indirect.

## Discussion

The basic structural unit (C_5_ conformation) of the 2.0_5_-helix has been mostly observed in short peptide structures containing noncoded amino acids^15^. Here we show that this conformation is widespread in protein structures, though the backbone geometry is relatively more folded (residue i + 1 in Fig. 1c) as compared to the fully-extended form observed when it is part of 2.0_5_-helix^15^. Moreover, the intra-residue N–H∙∙∙O=C hydrogen bonding in the C_5_ conformation, in conjunction with another inter-residue N–H∙∙∙N interaction, give rise to a fused-rings structure, which has been observed not only in a short peptide^16^, but also, as identified here, abounds in proteins. Although, the N–H∙∙∙O hydrogen bond geometry, when the N–H and C=O groups of the same residue interact, is significantly away from ideal values, it is likely that there is cooperativity in the formation of the two C_5_-interactions, conferring considerable stability to the peptide and protein structures.

ϕ, ψ angles centered around −90° and 0° constitute the “bridge region” of the Ramachandran plot^29^, and are generally considered unfavorable due to a steric repulsion between the peptide nitrogen (N_i_) and the hydrogen of the next peptide unit (N_i+1_–H)^30^,^31^. Following the experimental confirmation to the contrary^10^ we observe that the occurrence in the region is rather widespread and results from the stability of the N–H∙∙∙N(p_z_) hydrogen bond. The possibility of participation in hydrogen bonding (within the protein or to solvent) has been suggested as the reason why the bridge region, disfavored under the unfolding conditions, becomes accessible in the folded structure^32^. However, the hydrogen bonds considered were of the type N–H∙∙∙OC, typically observed in β-turns and not N–H∙∙∙N(p_z_), identified here. The later type of interaction occurring between two adjacent peptide groups, in principle, should also be possible in the unfolded state, and has indeed been seen in isolated peptide structure^16^. The potential contribution to the stability due to the existence of N–H∙∙∙N(p_z_) interaction makes the bridge region rather populous in the folded state (and may also be so in the unfolded state), contrary to the earlier suggestion^32^.

Fused rings can be part of well known structural motifs, such as β-turns (Fig. 3a), Schellman motif (Fig. 3e). While Schellman and α_L_ are the two primary capping motifs at helix C-termini^33^, we find that a sharper turn (stabilized by the fused-rings motif) ending helix and linking it to a β-strand could be yet another capping motif (Fig. 3b), which essentially constraints the orientation between the two secondary structural elements. In a way this could be considered as an example of supersecondary structure mediated by the fused-rings motif. Fused-rings structure can also be recognized in another known motif, *viz*, β-bulge^23^. However, the latter almost exclusively occurs between antiparallel β-strands (Fig. 3d), whereas in our case it is not restricted to strands, and even when two such motifs are located in adjacent strands the latter are usually parallel (Fig. 5b).

The fused-rings motif is generally located between two β-strands, or at the beginning of a β-strand (Fig. 2c), examples can be seen in Figs 3a,b,e, 4 and 5a. Although helix capping motifs have been identified^33^, no comparable capping motif is reported in connection with β-sheet. The fused-rings motif may constitute a capping motif at the N-terminus of β-strand, where the hydrogen bond potential of the main-chain atoms of the first residue in the strand (or the residue prior to it) can be satisfied locally (as the *i*th residue of the fused-rings motif). Interestingly enough, whereas helix capping (at N-terminus in particular) involves the side chain^20^,^33^, the interaction here involves only the main-chain atoms–the fused-rings motif seems to be a self-sustaining motif where the side chain does not have specific role. In fact, its absence (in the form of Gly being preferred at position i) is a very prominent feature. As already noted, Gly is an important residue that may signify helix termination^34^,^35^; here we find Gly in a motif that occurs preceding β-strand.

Yet another motif which has relevance to our newly identified motif is the “nest”, in which two consecutive residues with enantiomeric backbone torsion angles (the two sets of ϕ, ψ angles are close to (−90°, 0°) and (+90°, 0°), or the other way round) are involved in harboring an egg–an anion or a moiety carrying a partial negative charge^36^,^37^. It may be mentioned that the NH group, especially of Gly, is preferred at the anion binding site in proteins^38^. This is also the residue of choice at the position i of fused rings, which again is found to adopt either of the conformational angles mentioned above. It is no wonder then the fused-rings motif can also constitute a “nest”. The existence of fused five-membered rings provides a holistic perspective to “nest” relating it to β-turns, Schellman motif and β-bulge, and is a common theme running through all these diverse local structures.

Last, but not the least, we have identified a new secondary structure, *topi*, with distinct shape that can link two fused-rings motif occurring distant from each other in primary structure (Fig. 5). Moreover, the fused-rings motif, even when it occurs in isolation in loop region, retains its distinct shape. Taken together with other recent observations on the existence of distinct geometry in ‘irregular’ regions^39^ would pave the way for structural characterization of loops in proteins. The majority of the peptide segments involved in *topi* are predicted to have tendency to protein aggregation, and may thus contribute to the pathogenesis of human disease-related proteins.

## Conclusion

The work shows how the intermingling of ideas between designed peptide and protein structures can lead to the identification of new motifs. Starting with the observation of fused-rings motif in a peptide structure, we find that the structure is rather wide spread in proteins (Fig. 1c). In 36% cases the N–H group in the motif partakes in additional short-range hydrogen bond and the structure can be component of well-defined β-turns and their mimics, such as Asx-turn or Ser/Thr turn, though the existence of the fused-rings was never reported in these structures. In the remaining cases the motif occurs isolated, with the N–H group exhibiting, if at all, a long range hydrogen bonding. Some of these, especially when the hydrogen bond connects the β-strand containing the motif with an antiparallel strand, it gives rise to the well-known β-bulge. However, in 686 cases (in 4114 protein chains), two such motifs, distant to each other in sequence, can be hydrogen bonded, taking the shape of a *topi* (Fig. 5). It would be of interest to see how two distant regions in protein can recognize each other to be spatially close, and more importantly, assume a regular shape. Loops, constituting non-regular region in protein structures, are recalcitrant to geometrical characterization^40^. The fused-rings motif, alone or in pairs (as *topi*) provide regularity in conformation in regions beyond the regular secondary structures and should make the loop conformation less intractable.

## Methods

A non-redundant dataset of 4114 protein chains present in 3976 PDB (Protein Data Bank)^41^, files were selected using PISCES server^42^, such that the resolution ≤2 Å, R-factor is ≤0.2, and the sequence identity between any two protein sequences is ≤25%. The files used are given in^7^. REDUCE^43^ was used to fix the hydrogen positions in the structures.

**Figure 7:**
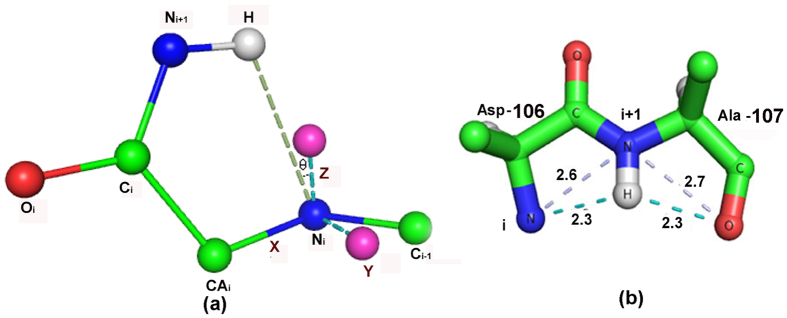
(**a**) The local axial system, indicating the directions of the three axes, used to define the angle (θ) subtended by the H···N_i_ direction with the z-axis passing through and perpendicular to the peptide plane containing N_i_. (**b**) Schematic representation of the two-residue motif (PDB file, 1A3C). The two hydrogen bonds and the associated distances (Å) are shown in dashed lines (cyan).

The directionality of the N–H group (at i + 1) towards the lone pair of electrons of the preceding N atom (at i) was measured considering a local axial system (Fig. 7a). N_i_ atom was placed at the origin, x-axis was along the N_i_–CA_i_ bond; z-axis was placed perpendicular to the peptide plane containing two bonds, N_i_–C_i-1_ and N_i_–CA_i_; y-axis completed the right-handed coordinate system. We then measured the angle (θ) between the N_i···_H_i+1_ direction and the z-axis. Only the cases with θ ≤ 30° were retained. Having identified one ring with C_5_ conformation, we identified the cases where this was part of fused-rings motif (Fig. 7b), consisting of two adjacent residues such that the main-chain NH group of one residue (at i + 1) forms two hydrogen bonds, one with the main-chain N atom (at i) and another with its own carbonyl O atom. The distances from N_i+1_ donor atom to the amide N (at i) and the carbonyl O (at i + 1) were restricted to be within 3 Å.

Additional hydrogen bond outside the motif was found out using HBPLUS^44^. The secondary structures were identified using DSSP^45^. Structural designations G (for 3_10_-helix), H (α-helix) and I (π-helix) were grouped as helix; B (β sheet) and E (extended strand) as β-strand; T (turn) and S (bend) as turn; and the remaining cases as belonging to irregular region (C) in the structure. The molecular diagrams were made using Pymol^46^. The residue preferences for each position of the fused-rings motifs along with the preceding residue, as part of β-turn mimic, were determined from sequence logos made using WebLogo 3 server^47^.

Statistical significance of the frequency of occurrence at the two positions of fused-rings motifs were calculated based on *z*-value^20^.


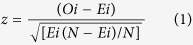


where *N* is the total number of fused-rings motifs, *Oi* is the number of observation of amino acid at position i, and *Ei* is the expected number. If l*z*l ≥ 1.96 (5% significance level), the observed number of occurrences was considered to deviate significantly from its expected value.

We calculated the sequence variability at i and i + 1 positions of *topi* in terms of entropy^48^. Shanon entropy at each position in the motif was calculated by considering multiple sequence alignment of all possible homologs of the protein (sequence identity ≥60%) using HSSP^49^ (homology-derived secondary structures of proteins) database. A residue in *topi* was considered as conserved if its entropy was less than the average entropy of the protein chain.

We used the statistical mechanics algorithm, TANGO ( http://tango.crg.es//)^28^, to find out if the sequences in the *topi* motif are prone to protein aggregation. It considers different competing conformations (β-turn, α-helix, β-sheet, the folded state and β-aggregates) and different energy terms, taking into account hydrophobicity and solvation energetics, electrostatic interactions and hydrogen bonding.

## Additional Information

**How to cite this article**: Dhar, J. *et al.* A novel secondary structure based on fused five-membered rings motif. *Sci. Rep.* **6**, 31483; doi: 10.1038/srep31483 (2016).

## Supplementary Material

Supplementary Information

## Figures and Tables

**Table 1:** Number of occurrences with increasing number of hydrogen bonding involving the N–H group.

Sl no	Type	Number
1	Single ring with N–H···N(p_z_) interaction (Fig. 7a)	77,388
2	Type 1, with additional hydrogen bond involving N–H (Fig. 1a)	49,266 (38,031)^a^
3	Fused-rings (Type 1, and hydrogen bond between N–H and C=O of the same residue) (Fig. 1c)	41,440
4	Fused-rings with additional short-range hydrogen bonding (Fig. 1d)	15,125^b^^,^^c^

^a^The number where the hydrogen bond is short-range (within four residues of the N–H group) is given in parentheses.

^b^Common between type 3 and short-range interaction of type 2. The number is 6,802 if one considers long-range interaction. 78 are hydrogen bonded to ligands and 3970 to water molecules.

^c^The range and the number of additional short range hydrogen bond interactions are given in Table 2.

**Table 2:** Occurrence of additional short range hydrogen bond interactions (beyond the fused-rings motif) involving the N-H group (Fig. 1d).

|D|^a^	Number^b^ of cases, with D	
	−ve	+ve
1	1 (1, 0)	32 (0, 32)
2	91 (75, 16)	2202 (0, 2202)
3	381 (90, 291)	10745 (10530, 215)
4	42 (13, 29)	1598 (900, 698)

^a^D is the difference in residue numbers (residue providing the N–H group–hydrogen bonding partner).

^b^In parentheses, the number of cases with hydrogen bonding involving the main-chain and side-chain atoms are given separately.
