# Supplementary material for: A novel secondary structure based on fused five-membered rings motif
Source: Sci Rep. 2016 Aug 11;6:31483. doi: 10.1038/srep31483 (PMC4980606; doi:10.1038/srep31483)
Supplement: Supplementary Information [file srep31483-s1.pdf]

A novel secondary structure based on fused five-membered rings motif

Jesmita Dhar,[1] Raghuvansh Kishore,[3*] and Pinak Chakrabarti[1,2,*]

[1] Bioinformatics Centre, [2] Department of Biochemistry, Bose Institute, P1/12 CIT Scheme VIIM, Kolkata 700054, India

[3] Protein Science & Engineering Division, CSIR-Institute of Microbial Technology, Sector 39-A, Chandigarh 160 036, India

***For Correspondence:**
(PC) E-mail: PC: pinak@jcbose.ac.in
Telephone: +91-33-2569-3253; Fax: +91-33-2355-3886
(RK) E-mail: kishore@imtech.res.in
Tel: +91-172-6665263; Fax: +91-172-690585

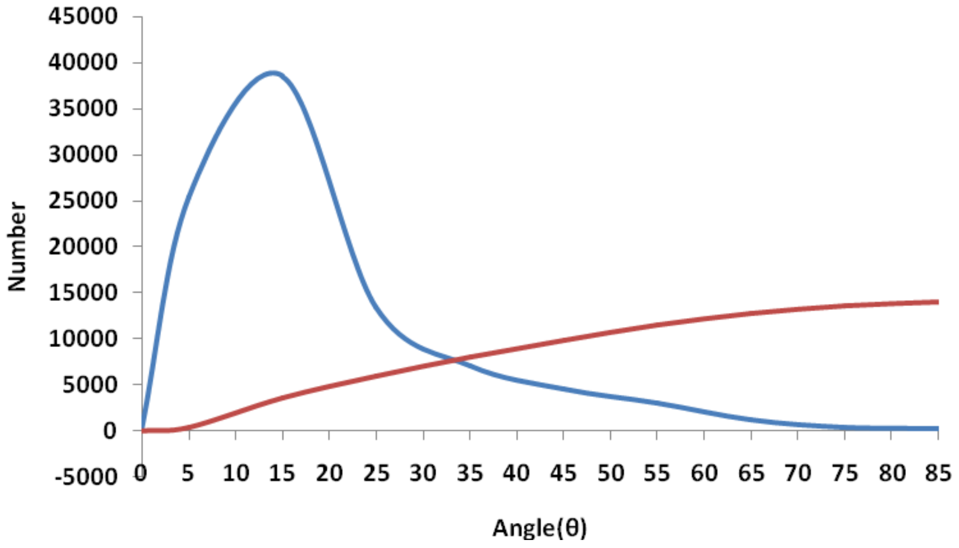

Fig. 1. Distribution of the angle θ (°) (blue) vs. the expected distribution (sine function, red).

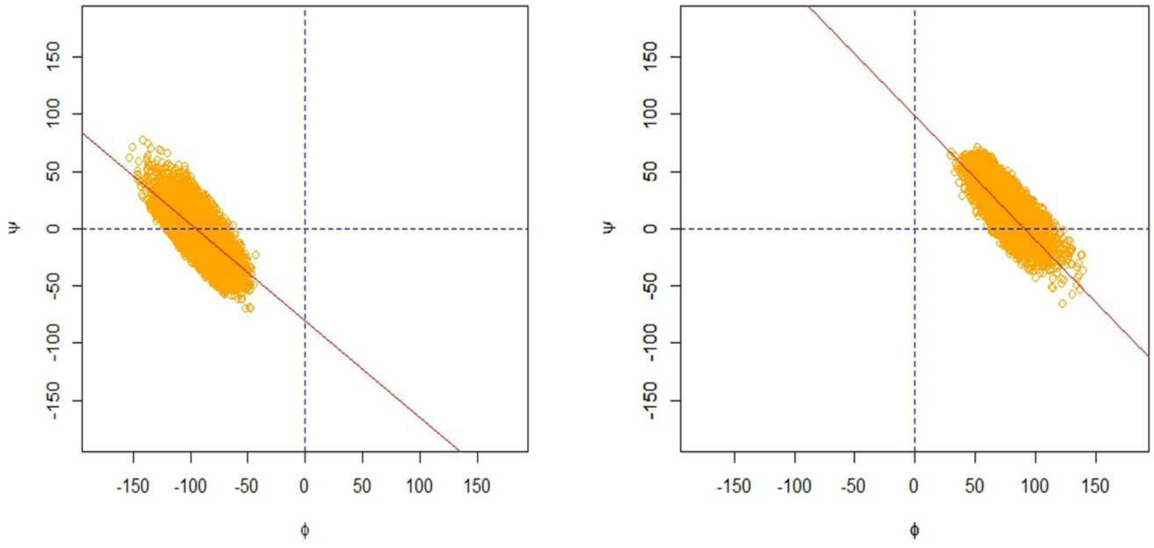

(a)

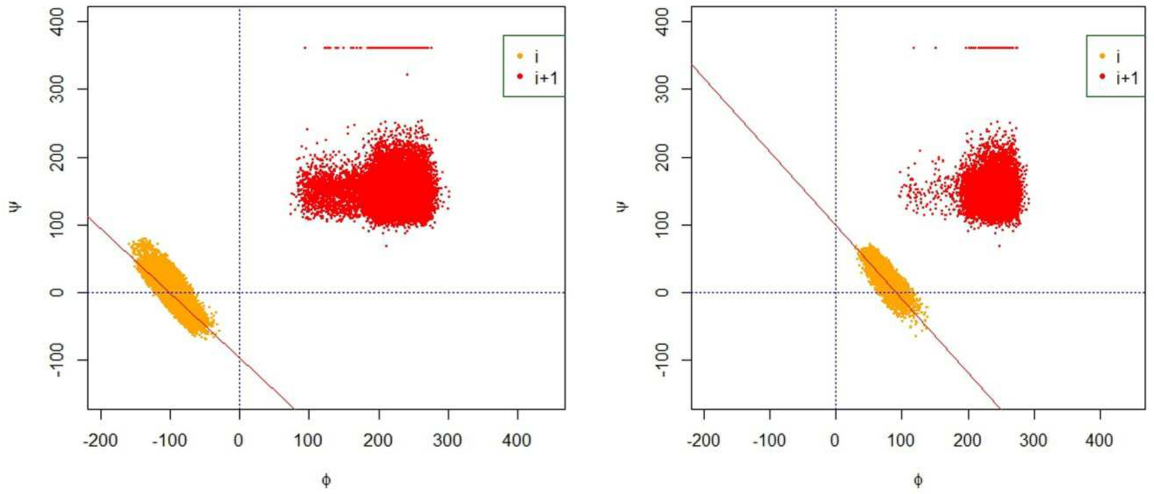

(b)

Fig. 2. Distribution of ϕ, ψ (°) angles of residues (a) i for 77,388 cases with N−H···N(p_z) interactions, (b) i (orange) and i+1 (red) for 41,440 cases with fused rings. The regression line passing through the data points for residue *i* is shown. The range of the axes has been altered in (b) to have a contiguous distribution of points.

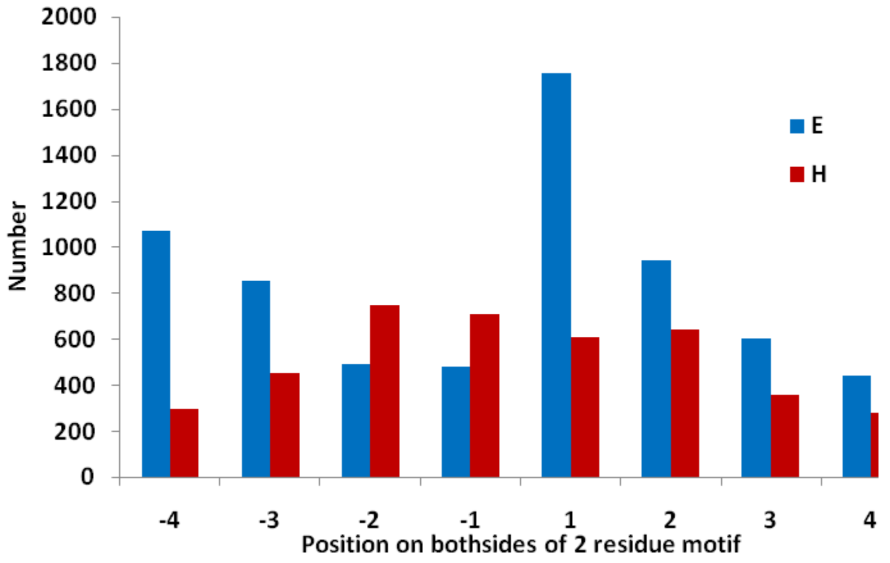

(a)

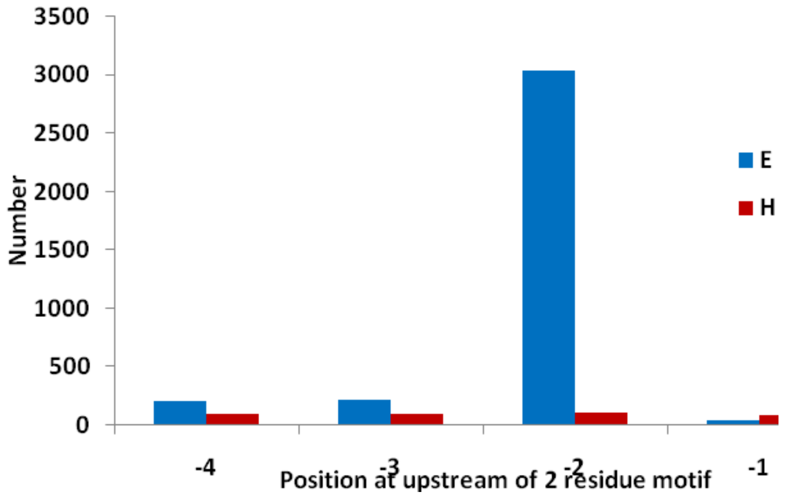

(b)

Fig. 3. The first occurrence of secondary structures (H or E) on either side of the fused-rings motifs (participating in additional short range hydrogen bond). This is same as Figure 2(c), except that the secondary structure combination of the fused-rings motif is C/C in (a) and C/E in (b).

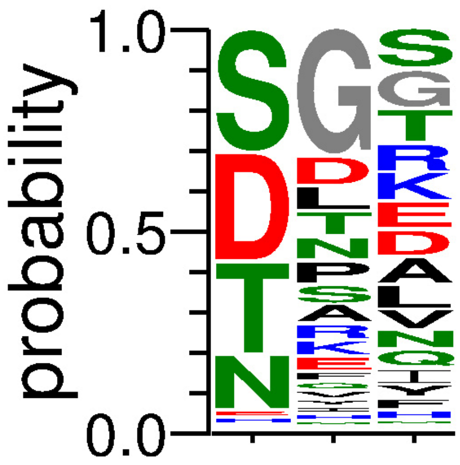

Fig. 4. The WebLogo for the for three-residue peptide stretch with the N−H group of the fused rings having additional short range interaction with a residue two residues preceding (*i.e.*, D = 2).

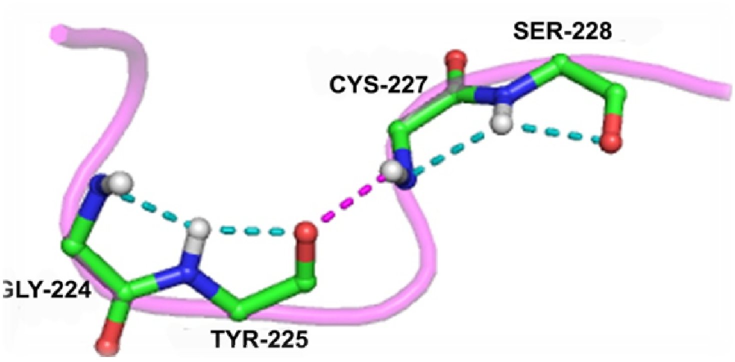

Fig. 5. Two linked fused-rings motifs (PDB, 1RTQ) with only one intervening residue. The interaction between these two motifs is shown by pink broken line.

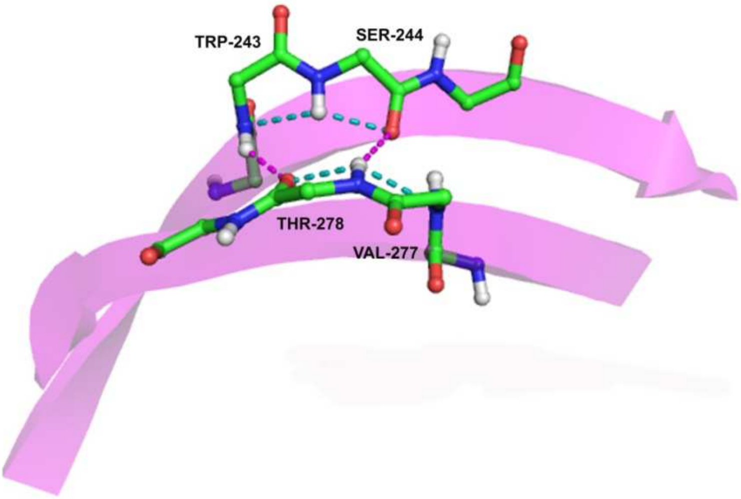

Fig. 6. Linked fused rings motifs (PDB, 2BLN) showing β-bulge type interaction.

Table 1. Propensity and *z*-value for the occurrence of residues at i and i+1 positions of fused-rings.

Amino acid	Position i		Position i+1	
	Propensity	*z*-value[a]	Propensity	*z*-value[a]
S	1.01	0.63	1.25	13.59
C	0.44	-12.44	1.10	1.91
M	0.50	-13.47	0.89	-2.74
E	0.83	-9.50	0.82	-9.07
Q	0.95	-1.72	1.04	1.53
K	1.09	4.16	1.18	9.18
R	0.95	-1.78	1.04	2.24
L	0.59	-26.00	0.86	-8.81
D	1.65	33.60	0.71	-15.10
N	2.09	47.62	0.91	-4.12
H	0.93	-2.37	0.98	-0.44
F	0.59	-16.83	1.31	12.93
Y	0.66	-13.22	1.35	14.30
W	0.59	-10.10	1.00	-0.15
V	0.39	-33.62	1.33	17.68
I	0.36	-31.07	1.25	12.66
T	0.88	-6.05	1.42	21.19
A	0.63	-22.54	0.67	-19.78
G	2.80	103.65	1.05	3.40
P	0.81	-9.04	0.00	-45.05

[a] $Z > 1.96$ are given in red; $Z < -1.96$ are in green.

Table 2. Cases where the *i*th residue[a] in the fused-rings motif is part of β-turn

D[b]	Number of cases interacting with the main-chain	Major neighbouring secondary structure (# cases)	β-turn type and % of occurrence[b]
3	10530	EE(2745)	I′(54), II′ (23), I (16), II (6)
		HE(762)	I (89)
4	900	HE(94)	I (84)
-	26315[c]	-	I(10), II (7), I′ (2), II′ (0.3)

[a] To be considered as β-turn, the residue and its preceding one should have backbone angles expected for the two central residues of a β-turn type.
[b] Cases with D = 2 have interactions only with the side-chain atoms and are not part of any β-turn.
[c] Fused rings motif having no additional short range hydrogen bond interactions.

Table 3. List of linked fused-rings motifs. The residues i and i+1 of motif 1, and m and m+1 of motif 2 are provided, along with the residue number of the first position. It is assumed that the hydrogen bond donor is located on motif 1 and acceptor on motif 2. The cases are grouped into four categories depending on which residue of motif 1 forms hydrogen bond with which residue of motif 2.

PDB	Chain	Motif 1 (N−H donor)	Motif 2 (CO acceptor)	Orientation[a]
**(a) (i+1) → m (170)**				
1A3C	A	151G-K	135L-V	**P**
1B5E	A	213G-S	168R-S	**P**
1BYI	A	73P-Y	38P-V	**P**
1EJD	A	253N-A	278D-W	**I/A**
1EZG	A	74T-A	62N-T	**P**
1G5H	A	412L-F	356K-V	**P**
1H1N	A	5Q-W	268T-G	**P**
1I1W	A	85L-V	128V-V	**P**
1I1W	A	231P-E	202D-G	**P**
1K75	A	155K-K	125A-S	**P**
1K77	A	71W-G	63P-G	**I/A**
1M7J	A	346M-M	286V-A	**I/A**
1OF8	A	319I-N	337G-V	**I/P**
1OFL	A	120L-V	145Y-I	**P**
1OFL	A	145Y-I	176V-I	**P**

1OFL	A	176V-I	217G-I	**P**
1OFL	A	322A-L	354A-I	**P**
1OGO	X	323M-W	349T-M	**P**
1OGO	X	456I-I	491L-F	**P**
1OGQ	A	158G-N	183R-N	**P**
1OGQ	A	183R-N	206R-N	**P**
1OGQ	A	253N-N	277F-N	**P**
1PXZ	A	194N-H	225N-A	**P**
1R6D	A	120G-R	78D-A	**P**
1QG8	A	91E-Y	3K-V	**P**
1RU4	A	297G-F	320N-Y	**P**
1RYI	B	196N-H	6E-A	**P**
1TOA	A	216L-Q	198A-H	**I/P**
1UA4	A	337K-R	287Y-S	**P**
1UWS	B	443L-L	427L-A	**I/P**
1VL1	A	179L-Y	127D-L	**P**
1X38	A	394N-G	538D-A	**I/A**
1XCR	A	51K-G	165G-Q	**I/A**
1YAC	A	106K-Q	12D-A	**I/P**
1Z6M	A	25K-V	58P-V	**P**
1ZR3	A	268K-F	210D-A	**P**
2C2P	A	116R-I	27T-L	**P**
2CHO	A	128R-Y	367S-G	**P**
2CXN	A	410V-Q	480T-K	**P**
2CYJ	A	73M-L	68T-G	**I/A**
2D5W	A	57L-A	44F-I	**I/P**
2DKJ	A	229R-G	224T-H	**I/A**
2DKO	A	112S-S	44M-G	**P**
2DVM	A	277V-I	301E-I	**I/P**
2DY0	A	155I-I	127D-L	**P**
2EPL	X	85E-D	367K-E	**P**
2FB6	A	37K-H	5D-K	**P**
2FCJ	A	33N-G	11E-G	**I/A**
2G1P	A	48S-R	29E-C	**P**
2G3W	A	100R-E	79D-L	**P**
2G40	A	204R-R	148D-F	**P**
2G8S	A	192G-I	161G-K	**I/A**
2GJ4	A	162E-F	277R-V	**P**
2GJL	A	15E-H	39G-G	**I/P**
2GNP	A	75E-S	299N-H	**P**
2HLJ	A	66L-H	104L-A	**I/A**
2I4L	A	400P-W	347R-V	**P**
2INU	A	125F-G	182D-F	**P**
2INU	A	246G-N	224E-H	**P**
2IXD	A	99K-L	5H-I	**P**
2NOO	A	50L-A	37L-V	**I/P**
2NTP	A	114T-I	156A-L	**P**
2NTP	A	156A-L	179T-L	**P**
2NTP	A	179T-L	200F-I	**P**
2NTP	A	200F-I	231L-T	**P**
2NXV	A	76R-Y	17M-F	**P**
2O6S	A	84G-N	108T-N	**P**
2O6S	A	108T-N	132T-N	**P**

2O6S	A	132T-N	156Q-N	**P**
2O6S	A	156Q-N	180D-N	**P**
2OKT	A	175S-L	198P-F	**I/A**
2OLO	A	198G-K	6D-V	**P**
2OOK	A	109G-E	85E-R	**P**
2OSV	A	243F-T	224F-H	**P**
2OZT	A	169S-W	196P-L	**I/P**
2QJJ	A	214R-Y	238C-T	**I/P**
2RKQ	A	36P-R	105G-S	**P**
2UVJ	A	183P-V	237V-M	**I/P**
2V0H	A	364L-T	347F-V	**P**
2V3I	A	281T-L	273F-Y	**P**
2VBK	A	223V-G	255N-I	**P**
2VBK	A	419N-S	451G-V	**P**
2VBK	A	434G-I	467S-I	**P**
2VBK	A	373E-T	395Q-L	**P**
2VBK	A	297A-Y	319G-L	**P**
2VBK	A	319G-L	348G-I	**P**
2VBK	A	348G-I	382A-V	**P**
2VBK	A	382A-V	405G-V	**P**
2VBK	A	405G-V	434G-I	**P**
2VBK	A	467S-I	490A-V	**P**
2VFO	A	305I-I	333S-A	**P**
2VFO	A	333S-A	361G-V	**P**
2VFO	A	361G-V	393G-F	**P**
2VFO	A	393G-F	440G-F	**P**
2VFO	A	440G-F	462G-A	**P**
2VFO	A	462G-A	489Q-I	**P**
2VFO	A	489Q-I	515T-I	**P**
2VUW	A	759K-T	620S-S	**P**
2W7Z	A	36Q-I	56H-L	**P**
2W7Z	A	71N-V	91R-V	**P**
2W7Z	A	91R-V	111D-C	**P**
2W7Z	A	111D-C	131L-V	**P**
2WHL	A	265L-S	253W-K	**I/P**
2WMK	A	958G-I	987H-M	**I/A**
2WNH	A	283R-W	90S-I	**P**
2X5O	A	206K-V	172V-A	**P**
2X6W	A	342T-V	370S-V	**P**
2X6W	A	370S-V	391G-A	**P**
2X6W	A	391G-A	424F-V	**P**
2X6W	A	424F-V	464F-I	**P**
2X6W	A	464F-I	501A-I	**P**
2X6W	A	559L-F	586C-I	**P**
2XHG	A	410A-L	364E-V	**P**
2XT2	A	53E-C	73E-V	**P**
2Y24	A	297L-L	291I-R	**P**
2Y53	A	460G-R	436L-V	**P**
2YEQ	A	81G-L	21D-S	**I/A**
2YEQ	A	324H-W	270L-A	**P**
2YEQ	A	373N-V	401F-G	**P**
2Z0D	A	57N-F	67T-S	**I/P**

3DKR	A	86A-K	15D-T	**P**
3DLQ	R	190G-L	133R-S	**I/A**
3EU3	A	58P-V	92D-V	**P**
3EUR	A	29E-Y	64K-L	**P**
3F4S	A	40P-I	74K-M	**P**
3FF1	A	327N-H	381P-Q	**P**
3FO3	A	207K-W	195D-W	**I/P**
3FRH	A	125A-S	105R-R	**P**
3G5T	A	62E-Q	38K-L	**P**
3H2Z	A	62G-V	44H-S	**I/A**
3H74	A	12A-V	41S-T	**P**
3HBN	A	227N-K	159D-F	**P**
3ILX	A	148L-N	121A-Y	**P**
3IPF	A	30G-V	68G-Q	**I/P**
3JRN	A	64R-F	10D-V	**P**
3K6M	A	200E-T	167D-F	**P**
3KS3	A	140G-L	206C-V	**I/P**
3LHI	A	186G-H	128D-V	**P**
3M8U	A	74L-A	60L-V	**I/P**
3MD7	A	235K-R	204D-V	**P**
3MDW	A	35D-A	18N-V	**P**
3MQD	A	380N-V	353N-F	**I/A**
3N1M	C	779H-L	727T-T	**I/A**
3N6Z	A	206G-V	170G-Y	**P**
3N6Z	A	243G-R	206G-V	**P**
3N6Z	A	273G-Q	243G-R	**P**
3N6Z	A	285N-S	316N-C	**P**
3NRE	A	205G-W	230P-C	**I/A**
3O83	A	155E-I	127K-L	**P**
3OQI	A	60Q-S	30R-H	**P**
3PB6	X	160A-R	214P-V	**P**
3PG6	A	710T-S	719Y-G	**P**
3PZS	A	12V-F	41S-N	**P**
3QPA	A	33D-V	67G-V	**P**
3QUF	A	269G-V	186A-F	**P**
3R9P	A	363P-I	339G-I	**I/P**
3SUV	A	160E-Y	434Y-G	**P**
3UR8	A	324M-F	307L-F	**P**
3VII	A	462I-Y	446L-M	**P**
3VMV	A	203N-L	225D-I	**P**
3VMV	A	225D-I	248N-V	**P**
3VNY	A	244A-E	290S-C	**P**
4A35	A	258R-W	282P-T	**I/P**
4AQ4	A	162K-C	240D-C	**P**
4BA1	A	243K-G	222D-A	**I/A**
4DWD	A	201G-Y	225P-V	**I/P**
4EQB	A	230A-I	168S-I	**P**
4EU9	A	162E-K	130N-I	**P**
4EXK	A	223A-M	146A-L	**P**
4GFI	A	179G-W	203P-L	**I/A**
**(b) i →m (337)**				
1AYO	A	35I-G	5P-F	**I/A**
1B5E	A	168R-S	148C-T	**I/A**

1BF2	A	687G-W	664Y-A	**I/A**
1CL1	A	305L-F	193T-G	**I/A**
1CVR	A	180T-K	143G-Q	**P**
1CVR	A	404N-E	389V-G	**I/A**
1CZN	A	139V-G	132A-V	**I/A**
1D5T	A	278K-Q	6D-V	**P**
1D5T	A	390I-D	290R-V	**I/A**
1DC1	A	201N-I	212D-I	**I/A**
1E19	A	290E-R	229D-I	**P**
1E2W	A	215I-V	228L-T	**I/A**
1ES9	A	95K-I	40E-V	**P**
1EZG	A	38N-T	26V-T	**P**
1EZG	A	50Q-T	38N-T	**P**
1EZG	A	62N-T	50Q-T	**P**
1FTR	A	174E-S	272G-V	**I/A**
1FTR	A	251L-N	195G-A	**I/A**
1GA8	A	124W-L	199G-V	**P**
1GK9	B	464Q-A	456A-N	**I/A**
1GXM	A	596R-F	631W-G	**I/A**
1HQ0	A	799T-L	787F-Y	**I/A**
1I1W	A	16V-Y	262V-G	**P**
1JR7	A	242R-Y	230V-F	**I/A**
1L6R	A	80F-F	68G-I	**I/A**
1M1Z	A	207T-W	190G-A	**I/A**
1MML	A	186T-R	86I-L	**I/A**
1N40	A	281A-D	385Q-R	**I/A**
1NLS	A	181I-W	87E-W	**I/A**
1OE4	A	219K-L	89E-V	**P**
1OFL	A	164H-C	202H-C	**P**
1OFL	A	202H-C	234S-N	**P**
1OFL	A	234S-N	258G-N	**P**
1OFL	A	253E-N	275H-Q	**P**
1OFL	A	354A-I	398F-F	**P**
1OFL	A	380Q-L	408K-N	**P**
1OGO	X	266Y-W	232S-I	**P**
1OI7	A	201E-A	146R-V	**P**
1P5D	X	436D-T	415K-G	**I/A**
1PMM	A	290R-D	268K-S	**I/A**
1PXZ	A	170D-C	194N-H	**P**
1QKS	A	149H-V	233V-A	**I/A**
1QKS	A	196G-Y	177G-Q	**I/A**
1QLW	A	33L-S	41A-H	**I/A**
1QW9	A	311W-T	100K-S	**I/A**
1R0V	A	141E-Q	247R-K	**I/A**
1R4X	A	676N-V	696R-S	**I/A**
1ROC	A	31L-E	3S-I	**I/A**
1RU4	A	146G-V	168N-T	**P**
1RU4	A	197R-N	230N-S	**P**
1RU4	A	287R-S	311N-N	**P**
1RWR	A	158G-Y	120S-N	**P**
1SG4	A	60G-V	105N-G	**I/A**
1SQ9	A	14A-H	386R-S	**I/A**
1UA4	A	352E-Y	337K-R	**I/A**

1UI0	A	115Q-I	35K-L	**P**
1UWK	A	100R-V	484T-W	**P**
1UWS	B	339G-Y	343G-C	**P**
1V7W	A	16T-R	3Y-G	**I/A**
1V82	A	221F-V	226R-Y	**I/A**
1VHU	A	101K-Y	34K-A	**P**
1W6S	A	452L-G	472R-F	**I/A**
1W8S	A	95N-C	79L-N	**I/A**
1WD3	A	108G-F	187S-T	**I/A**
1WDJ	A	140L-L	112K-A	**P**
1WDJ	A	166G-V	150R-A	**I/A**
1XV2	A	150D-I	119K-H	**I/A**
1YKI	A	88T-A	182K-G	**I/A**
1YT8	A	431E-R	392G-T	**P**
1YU0	A	193I-Y	181G-M	**I/A**
1Z45	A	604V-D	642P-T	**I/A**
1ZJC	A	298E-K	282Q-F	**I/A**
2AMH	A	34R-N	10R-T	**P**
2BJI	A	1254S-N	1209D-A	**I/A**
2C2P	A	27T-L	79G-L	**P**
2CDU	A	62Y-S	38S-F	**I/A**
2CF5	A	120S-Y	95C-L	**I/A**
2CH5	A	308R-H	334N-A	**I/A**
2CH5	A	343F-S	300S-S	**I/A**
2CHO	A	310T-G	282Y-N	**P**
2CNQ	A	123G-Y	214A-D	**I/A**
2D1S	A	149K-T	126T-I	**P**
2D1S	A	312V-E	285T-S	**P**
2D5W	A	245Q-K	6N-S	**P**
2DBN	A	175W-F	168F-E	**I/A**
2DSJ	A	373L-K	386L-A	**I/A**
2F1N	A	103N-R	38D-I	**I/A**
2FB5	A	115N-A	146V-S	**I/A**
2FCT	A	205I-D	169N-Y	**I/A**
2FNA	A	347S-E	336W-I	**I/A**
2FQX	A	184N-T	152G-M	**I/A**
2FRE	A	95D-H	152G-F	**I/A**
2FYG	A	54A-I	121G-M	**I/A**
2G0W	A	243R-V	195V-S	**P**
2G40	A	185G-G	128G-T	**I/A**
2GAI	A	34M-G	12E-S	**I/A**
2GAK	A	223I-K	397L-F	**I/A**
2H1V	A	249Q-A	175N-A	**P**
2HBA	A	6L-K	35L-A	**I/A**
2HIY	A	98A-R	140F-S	**I/A**
2HL0	A	19A-L	118G-Y	**I/A**
2HL0	A	37E-V	9D-Y	**I/A**
2I8T	A	38T-N	123Q-H	**I/A**
2ID4	A	595I-D	501N-F	**I/A**
2INU	A	222Y-L	244E-C	**P**
2INU	A	262G-N	286G-N	**P**
2INU	A	286G-N	309S-N	**P**
2INU	A	309S-N	333A-N	**P**

2INU	A	333A-N	371-D-N	**P**
2INU	A	371-D-N	405L-N	**P**
2IW1	A	39Q-S	8Y-K	**P**
2IXD	A	142E-S	99K-L	**P**
2IZR	A	192Y-I	157N-L	**I/A**
2J1V	A	91N-F	110N-N	**I/A**
2J6G	A	242N-Q	122V-G	**I/A**
2JDC	A	145I-T	104D-L	**I/A**
2JE8	A	640M-G	551R-F	**P**
2JE8	A	658S-I	647L-N	**P**
2NML	A	65A-D	10P-T	**I/A**
2NT0	A	79K-G	376V-G	**P**
2NTP	A	126S-L	170D-V	**P**
2NXV	A	203Y-G	84D-V	**I/A**
2O6S	A	84G-N	108T-N	**P**
2O6S	A	132T-N	156Q-N	**P**
2OA2	A	180F-R	281E-T	**I/A**
2ODF	A	81R-F	15A-V	**I/A**
2OV0	A	2K-A	62L-G	**I/A**
2OV0	A	83T-G	2K-A	**I/A**
2P1M	B	515S-C	554E-R	**I/A**
2PMA	A	24N-A	154N-G	**P**
2PYX	A	89Q-G	271S-C	**P**
2Q9U	A	224K-Y	165K-I	**P**
2QJV	A	9T-S	15I-Q	**I/A**
2QWU	A	151I-S	182G-L	**I/A**
2QYC	A	63V-D	37G-L	**I/A**
2R5O	A	324E-Y	290G-A	**I/A**
2UXQ	A	381I-K	366G-Y	**P**
2V0H	A	277N-V	259R-F	**P**
2V0H	A	347F-V	329F-S	**P**
2V3I	A	231T-T	255T-C	**I/A**
2V9K	A	364H-R	331K-A	**I/A**
2VBK	A	395Q-L	419N-S	**P**
2VBK	A	476F-V	499C-V	**P**
2VEC	A	212D-E	179P-R	**I/A**
2VFO	A	383D-S	430N-L	**P**
2VFO	A	430N-L	452N-I	**P**
2VFR	A	274H-F	106S-L	**P**
2VG0	A	78E-L	33R-H	**P**
2VPN	A	129D-E	180D-G	**I/A**
2VSM	A	214G-T	586N-V	**P**
2VY8	A	649V-R	634S-S	**I/A**
2W1Z	A	352S-R	337K-M	**I/A**
2W7Z	A	31N-H	51D-A	**P**
2W7Z	A	51D-A	71N-V	**P**
2WA2	B	226G-I	71G-V	**I/A**
2WBN	A	361D-A	286N-R	**P**
2WQK	A	173K-G	224G-Y	**I/A**
2WQP	A	334A-C	347D-I	**I/A**
2WVV	A	141L-W	133T-K	**I/A**
2WYH	A	824Y-L	503M-S	**I/A**
2X4J	A	44Y-N	80K-S	**I/A**

2X6W	A	320N-C	349Y-S	**P**
2X6W	A	349Y-S	377D-T	**P**
2X6W	A	446N-G	487N-A	**P**
2X6W	A	487N-A	515A-N	**P**
2X6W	A	569N-S	597S-D	**P**
2X6W	A	692E-I	670D-F	**I/A**
2XIO	A	27K-H	20G-I	**I/A**
2XT2	A	13G-E	33D-C	**P**
2XT2	A	73E-V	99A-L	**P**
2XWT	C	43D-I	61E-T	**P**
2XWT	C	250K-E	229S-L	**P**
2XWV	A	132N-R	183D-G	**I/A**
2Y24	A	45Q-G	284S-A	**P**
2YC3	A	173E-L	78S-V	**P**
2YHA	A	738E-N	657K-T	**P**
2Z5W	A	129S-E	117A-I	**I/A**
2ZM9	A	211G-E	161Q-I	**I/A**
2ZUV	A	353K-Y	331D-A	**P**
2ZUV	A	638V-N	564G-V	**I/A**
2ZWA	A	432S-S	478R-E	**P**
2ZZV	A	163K-K	213D-A	**I/A**
3A0Z	A	750K-T	681T-K	**I/A**
3ACH	A	90N-T	178R-D	**I/A**
3B0G	A	450I-I	480G-C	**I/A**
3B0X	A	351Q-N	531H-Q	**I/A**
3B0X	A	367R-Y	338K-G	**P**
3B5M	A	96K-R	81D-L	**I/A**
3B9T	A	316D-N	291G-T	**I/A**
3BIY	A	1431T-T	1391R-R	**P**
3BVX	A	935A-E	523Y-F	**I/A**
3BWS	A	276I-G	257E-D	**P**
3C85	A	37Q-V	61I-S	**P**
3C9H	A	229V-L	172R-I	**P**
3CBO	A	322L-R	302N-C	**I/A**
3CKC	A	470T-R	379G-D	**I/A**
3CLA	A	87D-S	7N-Y	**I/A**
3CLM	A	102G-F	38C-G	**P**
3CLM	A	280H-T	11G-Q	**P**
3CP7	A	63Y-G	139Q-E	**I/A**
3D06	A	237M-C	194L-I	**I/A**
3D1R	A	57E-G	66L-Y	**P**
3DA0	A	116A-A	164I-K	**I/A**
3DJL	A	80D-D	71R-Y	**I/A**
3DJL	A	242R-F	223D-A	**I/A**
3DT5	A	148Y-A	135K-A	**I/A**
3E3X	A	235F-I	248I-G	**I/A**
3E3X	A	252S-R	231R-G	**I/A**
3ETJ	A	122R-T	149E-C	**I/A**
3F9X	A	233T-K	217G-M	**I/A**
3FF1	A	261T-T	318F-L	**P**
3G5B	A	615C-R	571A-I	**I/A**
3G5B	A	675D-Q	660P-C	**I/A**
3G5B	A	703A-L	735G-G	**I/A**

3G7U	A	111L-F	71D-G	**P**
3GBW	A	1236R-F	1380P-Q	**I/A**
3GF6	A	219Q-K	148E-W	**I/A**
3GWQ	A	311A-L	280L-T	**I/A**
3GY9	A	109D-R	74R-T	**P**
3GYK	A	20D-V	50N-V	**P**
3H09	A	493A-F	513Q-V	**P**
3H20	A	173G-K	95A-L	**I/A**
3HID	A	426L-R	412D-I	**I/A**
3HKW	A	278R-C	3S-Y	**I/A**
3HO6	A	149K-N	104E-K	**P**
3HR6	A	158A-E	86G-M	**I/A**
3HRL	A	50Q-Q	59D-F	**I/A**
3HSU	A	217T-W	280K-S	**I/A**
3IJM	A	53E-T	66D-V	**I/A**
3IP4	B	27A-H	18D-S	**I/A**
3IT5	A	86Q-V	99L-G	**I/A**
3JU4	A	598T-R	578D-A	**I/A**
3KD4	A	55F-T	26E-A	**I/A**
3KG9	A	1031D-V	1195D-K	**I/A**
3KWE	A	143D-V	126N-A	**P**
3LAT	A	97t-A	86H-A	**I/A**
3LGD	A	258I-G	215L-Y	**P**
3LM4	A	75S-S	7D-I	**I/A**
3LS9	B	22D-A	8T-R	**P**
3LWG	A	83L-A	119V-F	**I/A**
3M0M	A	222M-Y	257H-H	**I/A**
3M1X	A	82A-d	118L-V	**I/A**
3MDM	A	88T-S	73P-V	**I/A**
3MDW	A	18N-V	7E-R	**I/A**
3MYX	A	119S-G	163A-S	**I/A**
3N6Z	A	150N-V	188N-C	**P**
3N6Z	A	188N-C	221N-C	**P**
3N6Z	A	254N-C	285N-S	**P**
3N6Z	A	306G-V	273G-Q	**P**
3N6Z	A	273G-Q	243G-R	**P**
3N6Z	A	243G-R	206G-V	**P**
3N6Z	A	206G-V	170G-Y	**P**
3NVS	A	265G-I	293D-Y	**I/A**
3NY3	A	117V-D	156A-W	**I/A**
3O3M	A	257K-Y	282G-V	**P**
3OEP	A	188L-L	178V-Y	**I/A**
3OG2	A	476D-Y	485L-Y	**I/A**
3OIZ	A	431D-R	402R-E	**P**
3OND	A	200V-G	134D-L	**P**
3OO8	A	354K-S	283D-Y	**P**
3OR1	A	187C-Y	220G-C	**I/A**
3P0Y	A	422T-S	391T-D	**P**
3P2E	A	25D-R	48N-T	**P**
3POP	A	33E-E	77D-L	**P**
3POP	A	347R-A	440R-T	**I/A**
3POW	A	158N-K	199D-W	**I/A**
3PZW	A	604L-I	590V-A	**I/A**

3Q2U	A	167N-G	157P-K	**I/A**
3R9F	A	135K-R	99K-T	**P**
3RM3	A	89Q-T	20P-V	**P**
3RPD	A	112V-G	89G-V	**I/A**
3RQT	A	49L-V	36L-F	**I/A**
3S5M	A	1049Y-G	1067R-D	**I/A**
3S5Q	A	92T-D	100S-S	**I/A**
3S5Q	A	133N-E	165I-V	**I/A**
3SBT	B	31H-G	101M-M	**P**
3SC7	X	372N-Q	506Q-S	**I/A**
3SGG	A	144K-G	171R-G	**P**
3SGG	A	448D-A	422N-I	**P**
3SGZ	A	69S-A	99N-I	**I/A**
3SIG	A	253A-H	215R-R	**P**
3SUV	A	204R-I	197L-S	**I/A**
3T7H	A	88N-V	36D-S	**I/A**
3TG0	A	43K-N	361N-T	**P**
3U2U	A	96S-K	3D-Q	**P**
3U65	A	144R-A	197D-G	**I/A**
3UD1	A	1142H-R	1099A-V	**I/A**
3UPL	A	359K-K	375C-Y	**I/A**
3UPS	A	48D-Y	97D-I	**P**
3UQS	A	32T-S	416L-W	**I/A**
3UR8	A	332K-Y	324L-F	**I/A**
3UWS	B	195E-Y	172D-F	**P**
3VKJ	B	59S-V	89G-I	**I/A**
3VKW	A	762A-W	801V-V	**I/A**
3VKW	A	926D-I	901K-R	**P**
3VMV	A	174N-H	203N-L	**P**
3VMV	A	198H-N	220N-N	**P**
3VMV	A	220N-N	243N-N	**P**
3VMV	A	109N-V	136H-N	**P**
3VXJ	A	32N-D	145S-I	**I/A**
3ZX4	A	85L-A	61G-G	**I/A**
4AT7	B	117K-E	88T-L	**I/A**
4B4P	A	18K-T	36N-V	**P**
4BA1	A	194N-Y	123A-L	**I/A**
4D9S	A	155G-D	213L-D	**I/A**
4D9S	A	207G-D	265L-I	**I/A**
4D9S	A	259G-D	317C-S	**I/A**
4D9S	A	311G-N	369N-F	**I/A**
4DLQ	A	791L-E	763N-S	**I/A**
4DN7	A	84K-D	68S-G	**I/A**
4DO4	A	124N-F	159F-S	**I/A**
4E2U	A	148N-N	74E-K	**I/A**
4EDG	A	200R-F	187L-L	**I/A**
4EHX	A	171R-E	44L-S	**I/A**
4EI0	A	123T-V	107V-C	**I/A**
4EIU	A	161G-V	136F-L	**I/A**
4EU9	A	315S-S	294E-V	**P**
4EVW	A	178N-R	131D-G	**I/A**
4EZG	A	107T-N	85T-D	**P**
4F27	A	412G-Y	472N-V	**I/A**

4F3M	A	130R-R	45I-Y	**I/A**
4F6T	B	269A-S	241R-E	**I/A**
4F9D	A	369W-F	355A-F	**I/A**
4FQI	B	141Y-H	129N-A	**I/A**
4FS7	A	103R-E	80V-S	**P**
4FS7	A	171T-V	149K-S	**P**
4FS7	A	262T-E	240Y-Y	**P**
4FS7	A	240Y-Y	217K-I	**P**
4FS7	A	217K-I	194E-T	**P**
4FS7	A	375R-K	352E-E	**P**
4FS7	A	352E-E	329K-F	**P**
4FS7	A	329K-F	306V-I	**P**
4G5A	A	61K-D	44W-L	**I/A**
4GAK	A	66H-Q	103L-A	**I/A**
4GMF	A	211R-Y	192D-F	**I/A**
4H7N	A	230L-G	435S-G	**I/A**
4HCJ	A	154N-G	143N-V	**I/A**
**(c) (i+1) → (m+1) (24)**				
1D4O	A	99D-L	23N-S	**I/P**
1D5T	A	106E-G	315S-C	**I/P**
1RGZ	A	46A-D	51N-K	**I/A**
1RGZ	A	51N-K	46A-D	**I/A**
1VHU	A	34K-A	140E-S	**I/P**
1XCR	A	53I-C	165G-Q	**I/A**
1XCR	A	165G-Q	53I-C	**I/A**
1ZR3	A	210D-A	304K-S	**I/P**
2AHF	A	335R-G	347D-D	**I/A**
2AHF	A	347D-D	335R-G	**I/A**
2BZV	A	241N-C	249D-A	**I/A**
2BZV	A	249D-A	241N-C	**I/A**
2JE8	A	704Q-M	727S-L	**I/A**
2JE8	A	727S-L	704Q-M	**I/A**
2RDG	A	26N-L	64N-L	**I/A(β-bulge)**
2RDG	A	64N-L	26N-L	**I/A(β-bulge)**
3BRC	A	95D-L	37R-R	**I/P**
3DK9	A	283D-C	188G-R	**I/P**
3N6Z	A	206G-V	169T-G	**I/P**
3N9K	A	244G-Q	239L-T	**I/A**
3P2C	A	156N-A	180H-E	**I/A**
3R5T	A	291L-G	262T-M	**I/A**
3VII	A	202A-S	210S-I	**I/A**
4A6R	A	386K-N	391R-E	**I/A**
**(d) i → (m+1) (155)**				
1ARB	A	242S-R	238G-A	**I/A**
1AYO	A	18D-G	15Q-T	**I/P**
1B25	A	107D-A	6G-R	**I/P**
1CCW	A	40L-S	13S-D	**I/A**
1ESG	A	54C-N	50T-E	**I/A**
1EVX	A	57N-G	60R-W	**I/A**
1EX2	A	149A-Y	145D-K	**I/A**
1F0N	A	144I-Y	119G-S	**I/P**
1F5V	A	161A-D	15R-H	**I/A**

1GWE	A	196E-M	192R-T	**I/A**
1HP1	A	151L-F	141N-I	**I/A**
1HX0	A	38G-G	11T-S	**I/A**
1K75	A	204D-K	125S-V	**I/P**
1KID	A	252E-D	224D-K	**I/A**
1KYF	A	857K-H	937Q-F	**I/A**
1L7A	A	198K-A	174R-I	**I/P**
1LQV	A	56L-Q	53L-Q	**I/P**
1LVW	A	84R-G	81E-E	**I/P**
1LZL	A	182A-F	153R-I	**I/P**
1NSZ	A	199K-D	203E-I	**I/A**
1NSZ	A	203E-I	199K-D	**I/A**
1OEW	A	10I-D	15D-A	**I/A**
1Q35	A	171A-R	132K-V	**I/P**
1R6X	A	304F-Y	295P-G	**I/A**
1RGZ	A	46A-D	51N-K	**I/A**
1RGZ	A	344N-T	26P-G	**I/A**
1RTQ	A	227C-S	224G-Y	**I/P**
1RU4	A	256G-I	230E-N	**I/A**
1RU4	A	297G-F	318G-I	**I/P**
1S0I	A	505l-D	510G-K	**I/A**
1UWS	B	343G-C	339G-Y	**I/A**
1V7W	A	613L-N	639G-A	**I/A**
1W96	A	91T-V	87G-D	**I/P**
1W96	A	144D-A	60S-K	**I/P**
1X38	A	478S-Y	411P-K	**I/P**
1YC5	A	182S-L	14R-L	**I/P**
1YE8	A	142V-H	139I-R	**I/P**
1YQZ	A	209N-E	179R-S	**A**
1ZZ1	A	259Q-L	172E-R	**I/P**
2ABW	A	95K-N	114G-L	**I/P**
2AJ7	A	40F-A	45P-Y	**I/A**
2B0T	A	342D-V	338H-V	**I/A**
2BHU	A	141G-T	137F-T	**I/A**
2BLN	A	277V-T	243W-S	**A(β-bulge)**
2BLN	A	243W-S	277V-T	**A(β-bulge)**
2BWR	A	293K-V	289L-T	**I/A**
2CDU	A	237D-I	150K-T	**I/A**
2CO3	A	67V-D	72T-A	**I/A**
2DBN	A	266L-S	357G-N	**I/A**
2DKO	A	112S-S	42P-E	**I/P**
2F1N	A	203T-Q	207Q-R	**I/A**
2F1N	A	207Q-R	203T-Q	**I/A**
2F9F	A	89K-G	14D-F	**I/A**
2FVV	A	27R-S	32E-E	**I/A**
2HL0	A	133A-E	129G-H	**I/A**
2HLQ	A	119S-T	95H-Y	**I/A**
2IKS	A	119D-A	62R-S	**I/P**
2IKS	A	240Q-A	180E-T	**I/P**
2INU	A	246G-N	222Y-L	**I/P**
2J43	A	221Y-I	217N-N	**I/A**
2JB7	A	59R-L	55A-S	**I/A**

2JCB	A	120D-L	45K-T	**I/P**
2JDC	A	104D-L	67Q-K	**I/P**
2JE8	A	210W-R	112E-G	**I/A**
2NRR	A	424P-N	361Y-R	**I/P**
2NTP	A	265L-G	301Y-G	**I/P**
2NXV	A	59F-H	56E-G	**I/P**
2OCT	A	59V-G	62E-D	**I/A**
2P1M	B	55A-V	51G-N	**I/A**
2Q9U	A	138I-G	140K-R	**I/A**
2RDG	A	109T-G	112H-T	**I/A**
2UVJ	A	138I-S	307A-Q	**I/A**
2UVJ	A	307A-Q	138I-S	**I/A**
2VG0	A	207D-L	33R-H	**I/P**
2W8T	A	55K-S	58T-E	**I/A**
2W91	A	650A-W	646D-D	**I/A**
2WAO	A	71A-T	50G-G	**I/A**
2WDC	A	75Y-L	71G-R	**I/A**
2WUU	A	417A-L	287D-V	**I/P**
2X5O	A	112S-N	177L-N	**I/A**
2X6W	A	370S-V	390R-G	**I/P**
2X6W	A	501A-I	521R-Y	**I/P**
2X6W	A	559L-F	584F-S	**I/P**
2XHA	A	138L-S	143E-Y	**I/A**
2XTS	A	362K-A	334N-G	**I/A**
2XWT	C	225Y-S	199G-T	**I/P**
2Y9F	A	94N-W	90R-Y	**I/A**
2YCL	A	77H-E	60P-V	**I/A**
2YCL	A	85D-K	81L-F	**I/A**
2ZUV	A	29D-A	6R-F	**I/P**
2ZWA	A	396A-G	398D-V	**I/A**
3B9T	A	261D-Y	258P-I	**I/A**
3BFM	A	111S-T	120D-W	**I/A**
3BON	A	398A-A	394N-T	**I/P**
3BWS	A	298A-S	303S-G	**I/A**
3BXP	A	148A-A	109R-I	**I/P**
3CBW	A	259Y-V	255P-G	**I/A**
3CKJ	A	301D-G	306Q-S	**I/A**
3CUZ	A	112K-V	91R-V	**I/P**
3DK9	A	248F-S	218R-H	**I/A**
3E4W	A	159T-T	155A-S	**I/A**
3EDF	A	239W-I	235P-T	**I/A**
3EW8	A	258K-A	170E-R	**I/P**
3FEG	A	295F-Y	291E-E	**I/A**
3FN5	A	209T-D	214T-E	**I/A**
3FOT	A	22T-G	27R-Q	**I/A**
3FRH	A	168D-L	105R-R	**I/P**
3FWZ	A	483K-W	418N-H	**I/P**
3G8Y	A	273Y-A	249R-I	**I/P**
3GF6	A	75G-K	219Q-K	**I/A**
3GZA	A	327N-A	424H-K	**I/A**
3H09	A	67V-L	53N-V	**I/A**
3H09	A	888K-V	884N-D	**I/P**
3HSU	A	38A-A	82G-H	**I/P**

3IOH	A	316T-K	227P-L	**I/P**
3JRN	A	64P-F	8K-Y	**I/P**
3JYO	A	196D-G	128D-S	**I/P**
3LED	A	119G-A	176D-A	**I/P**
3LEW	A	353R-D	358Q-L	**A**
3LEW	A	358Q-L	353R-D	**A**
3M0M	A	189Q-S	185N-F	**I/A**
3MDW	A	210A-V	206H-S	**I/P**
3MT0	A	88G-L	5R-S	**I/P**
3MU7	A	223A-A	219A-H	**I/A**
3NBC	A	65V-N	47Q-Q	**I/A**
3NT1	A	50T-G	53D-Q	**I/A**
3P2C	A	180H-E	156N-A	**I/A**
3PE7	A	231V-K	217R-M	**I/A**
3PIC	A	295V-L	271K-I	**I/P**
3PIJ	B	154D-N	159T-S	**I/A**
3POH	A	397G-I	424W-F	**I/P**
3QVM	A	123S-D	98N-V	**I/P**
3QVR	A	439L-D	446N-H	**I/A**
3RPD	A	321N-C	286A-I	**I/P**
3RVA	A	35S-G	67N-C	**I/A**
3RVA	A	67N-C	35S-G	**I/A**
3SM4	A	108R-D	113R-T	**I/A**
3SZU	A	215E-V	260K-C	**I/P**
3T6Q	A	330A-N	352I-K	**I/P**
3T6Q	A	356T-K	330A-N	**I/P**
3T6S	A	77S-L	138H-A	**I/P**
3U81	A	136D-M	60S-L	**I/P**
3UC1	A	619D-A	615R-G	**I/P**
3V5A	A	555E-S	551N-T	**I/A**
3VEN	A	43N-T	12D-F	**I/A**
3VGI	A	317L-I	153N-L	**I/A**
3VSS	A	350K-N	355T-E	**I/A**
4A5S	A	648K-C	623R-I	**I/P**
4AQ4	A	242A-M	162K-C	**I/P**
4DB5	A	105Q-V	101N-S	**I/A**
4DT1	A	209D-S	8L-I	**I/P**
4EQB	A	213K-A	168S-I	**I/P**
4EW7	A	66Y-N	62S-G	**I/A**
4FUS	A	343G-W	338A-D	**I/A**
4G0X	A	84D-L	12N-N	**I/P**

In the two minor categories (c) and (d) where the two motifs face each other (Figure 4), the hydrogen bond linking them may involve both the residues i and i+1, albeit with unequal H···O distances.

[a] P or A is used to indicate if the peptide segments containing the two motifs are parallel or antiparallel to each other. The letter is preceded by I (inclined) if the alignment of the segments is not very clear.

Table 4. Excerpts from TANGO output run on PDB files[a] where the *topi* motif (type (a) in Fig. 4) is observed. The NH donor and the CO acceptor are represented by blue and red colours, respectively.

Sl # PDB Chain Motif 1 Motif 2

Sequence Number	Residue name	% of β strand conformation	% of β turn conformation	% of α helical conformation	% of aggregation
1. 1A3C A	151G-K	135L-V			
151	G	0.5	0.8	0.219	0.000
152	K	0.6	0.8	0.219	0.000
135	L	7.6	0.0	0.673	93.264
136	V	7.0	0.1	0.563	92.936
2. 1B5E A	213G-S	168R-S			
213	G	0.2	1.0	0.000	2.939
214	S	0.7	1.0	0.000	1.281
168	R	1.6	0.4	0.000	0.000
169	S	0.8	0.8	0.000	0.000
3. 1BYI A	73P-Y	38P-V			
73	P	0.2	0.2	0.000	0.000
74	Y	11.0	0.2	0.000	0.000
38	P	0.2	0.0	0.000	0.000
39	V	2.3	0.3	0.000	0.000
4. 1EJD A	253N-A	278D-W			
253	N	0.6	0.4	0.000	0.000
254	A	0.1	0.4	0.000	0.000
278	D	0.3	0.6	0.000	0.000
279	W	0.9	0.4	0.000	0.000
5. 1EZG A	74T-A	62N-T			
74	T	1.1	0.0	0.000	0.000
75	A	0.7	0.0	0.000	0.000
62	N	0.8	0.2	0.000	0.000
63	T	6.8	0.2	0.000	0.000
6. 1G5H A	412L-F	356K-V			
412	L	2.4	0.0	1.388	96.742
413	F	6.3	0.0	1.114	97.770
356	K	9.1	0.0	0.137	0.568
357	V	12.2	0.0	0.137	0.568
7. 1H1N A	5Q-W	268T-G			
5	Q	10.5	0.0	0.000	3.052
6	W	6.7	0.2	0.000	3.052
268	T	2.2	0.2	0.120	20.677
269	G	0.3	0.2	0.120	22.301
8. 1I1W A	85L-V	128V-V			
85	L	3.2	0.0	0.000	0.000
86	V	4.3	0.0	0.000	0.000
128	V	7.6	0.0	0.000	0.996
129	V	5.9	0.0	0.000	0.996
9. 1I1W A	231P-E	202D-G			
231	P	0.0	0.2	0.000	0.000
232	E	1.6	0.2	0.000	0.000
202	D	0.7	0.9	0.000	0.000
203	G	0.7	1.1	0.000	0.000
10. 1K75 A	155K-K	125A-S			
155	K	0.4	0.3	0.000	0.000

156	K	2.2	0.1	0.000	0.000
125	A	2.7	0.0	0.000	0.971
126	S	3.1	0.0	0.000	1.164
11. 1K77 A	71W-G	63P-G			
71	W	0.1	0.2	0.256	0.000
72	G	0.2	0.2	0.256	0.000
63	P	0.1	1.8	0.000	0.000
64	G	0.0	1.8	0.000	0.000
12.1M7J A	346M-M	286V-A			
346	M	2.4	0.1	0.000	19.965
347	M	1.4	0.1	0.000	8.776
286	V	3.1	0.5	0.000	0.000
287	A	3.1	1.2	0.000	0.000
13. 1OF8 A	319I-N	337G-V			
319	I	1.9	0.5	0.000	0.000
320	N	1.8	0.9	0.000	0.000
337	G	0.4	0.2	0.000	2.169
338	V	3.9	0.0	0.000	2.169
14.1OFL A	120L-V	145Y-I			
120	L	0.9	1.0	0.475	66.803
121	V	4.0	0.0	0.475	67.875
145	Y	1.3	0.0	0.258	1.647
146	I	4.6	0.0	0.000	1.647
15. 1OFL A	145Y-I	176V-I			
145	Y	1.3	0.0	0.000	5.128
146	I	4.6	0.0	0.000	5.128
176	V	5.6	0.1	0.000	0.000
177	I	9.5	0.0	0.000	0.000
16. 1OFL A	176V-I	217G-I			
176	V	5.6	0.1	0.000	0.000
177	I	9.5	0.0	0.000	0.000
217	G	0.1	0.4	0.000	0.000
218	I	8.9	0.3	0.000	0.000
17. 1OFL A	322A-L	354A-I			
322	A	0.3	0.0	0.859	5.384
323	L	1.0	0.0	0.859	5.384
354	A	1.3	0.0	0.472	0.981
355	I	3.9	0.0	0.472	0.981
18. 1OGO X	323M-W	349T-M			
323	M	2.5	0.0	0.998	0.000
324	W	1.4	0.0	0.562	0.000
349	T	2.5	0.3	0.000	0.000
350	M	3.0	0.2	0.000	0.000
19. 1OGO X	456I-I	491L-F			
456	I	3.0	0.0	0.000	0.549
457	I	2.8	0.4	0.000	0.549
491	L	0.4	0.8	0.000	0.000
492	F	6.4	0.1	0.000	0.000
20. 1OGQ A	158G-N	183R-N			
158	G	0.3	1.1	0.000	0.000
159	N	0.1	1.0	0.000	0.000
183	R	1.3	1.1	0.102	0.000
184	N	0.3	1.1	0.440	0.000

21. 1OGQ A	183R-N	206R-N			
183	R	1.3	1.1	0.102	0.000
184	N	0.3	1.1	0.440	0.000
206	R	0.5	1.9	0.432	0.000
207	N	0.4	1.2	0.432	0.000
22.1OGQ A	253N-N	277F-N			
253	N	0.3	0.2	0.498	0.000
254	N	0.3	0.2	0.161	0.000
277	F	12.5	0.5	0.000	0.222
278	N	8.9	0.5	0.000	0.000
23.1PXZ A	194N-H	225N-A			
194	N	2.7	0.1	0.000	0.000
195	H	1.3	0.1	0.000	0.000
225	N	0.0	2.9	0.172	0.000
226	A	0.0	2.6	0.172	0.000
24.1R6D A	120G-R	78D-A			
120	G	0.1	0.3	0.000	0.000
121	R	2.0	0.3	0.000	0.000
78	D	0.7	0.0	0.000	0.124
79	A	1.2	0.0	0.000	0.124
25. 1QG8 A	91E-Y	3K-V			
91	E	0.1	0.7	0.000	0.000
92	Y	0.7	0.3	0.000	0.703
3	K	1.0	0.0	0.000	0.000
4	V	9.1	0.0	0.000	4.671
26. 1RU4 A	297G-F	320N-Y			
297	G	0.2	0.8	0.000	0.000
298	F	1.1	0.8	0.000	0.000
320	N	4.5	0.3	0.000	0.127
321	Y	4.5	0.2	0.000	0.127
27.1RYI B	196N-H	6E-A			
196	N	0.3	0.3	0.000	0.509
197	H	2.1	0.3	0.000	0.579
6	E	5.4	0.0	0.000	0.000
7	A	2.7	0.0	0.000	21.842
28. 1TOA A	216L-Q	198A-H			
216	L	0.1	0.5	0.000	0.000
217	Q	0.1	0.3	0.000	0.000
198	A	3.4	0.2	0.426	22.339
199	H	0.7	0.2	0.426	0.000
29. 1UA4 A	337K-R	287Y-S			
337	K	2.6	0.0	0.000	0.000
338	R	5.1	0.0	0.000	0.000
287	Y	7.3	0.0	0.000	49.466
288	S	7.0	0.0	0.000	34.406
30. 1UWS B	443L-L	427L-A			
443	L	0.4	0.2	0.000	0.250
444	L	1.8	0.2	0.000	0.250
427	L	1.9	0.4	0.109	0.000
428	A	0.1	0.6	0.109	0.000
31. 1VL1 A	179L-Y	127D-L			
179	L	1.3	0.2	9.504	85.202

180	Y	2.3	0.0	9.735	96.886
127	D	2.7	0.0	3.599	0.000
128	L	1.4	0.0	3.701	1.342
32.1XCR A	51K-G	165G-Q			
51	K	0.5	0.5	0.000	0.000
52	G	0.5	0.5	0.000	0.000
165	G	0.0	1.3	0.000	0.000
166	Q	0.0	2.6	0.000	0.000
33. 1YAC A	106K-Q	12D-A			
106	K	0.2	0.2	0.131	0.058
107	Q	0.4	0.1	0.131	1.300
12	D	0.0	3.9	0.371	0.000
13	A	0.1	0.2	0.371	16.560
34.1Z6M A	25P-V 58K-V				
25	P	0.0	0.0	0.000	0.000
26	V	7.5	0.0	0.000	7.881
58	K	6.5	0.3	0.238	0.000
59	V	7.8	0.1	0.238	0.000
35. 1ZR3 A	268A-K	210D-A			
268	A	0.2	0.0	0.000	0.000
269	K	0.6	0.0	0.000	0.000
210	D	0.1	3.6	0.180	0.000
211	A	0.7	3.6	0.180	0.000
36.2C2P A	116R-I	27T-L			
116	R	4.5	0.1	0.000	0.000
117	I	8.7	0.1	0.000	6.247
27	T	3.3	0.1	0.381	92.087
28	L	5.1	0.0	0.381	94.584
37. 2CYJ A	73M-L	68T-G			
73	M	0.7	0.0	0.200	20.769
74	L	2.3	0.0	0.200	19.197
68	T	0.1	0.6	0.000	41.530
69	G	0.1	0.6	0.000	38.205
38. 2DKJ A	229R-G	224T-H			
229	R	2.4	0.0	0.000	0.000
230	G	0.2	0.8	0.000	0.000
224	T	4.7	0.1	0.000	0.000
225	H	4.3	0.2	0.000	0.000
39. 2DKO A	112S-S	44M-G			
112	S	0.6	0.3	0.329	0.138
113	S	1.3	0.2	0.117	3.733
44	M	0.2	0.6	0.000	3.162
45	G	0.3	0.1	0.642	5.742
40. 2DVM A	277V-I	301E-I			
277	V	5.3	1.5	0.000	0.000
278	I	5.7	0.0	0.000	0.000
301	E	0.4	0.0	0.000	0.000
302	I	0.7	0.0	0.000	0.000
41. 2DY0 A	155I-I	127D-L			
155	I	2.5	0.0	0.310	68.457
156	I	6.4	0.0	0.310	68.457
127	D	0.3	0.1	0.406	0.000

128	L	0.2	0.1	0.406	0.955
42. 2FB6 A	37K-H	5D-K			
37	K	1.1	0.1	0.000	0.000
38	H	6.6	0.1	0.000	0.000
5	D	0.0	2.9	0.934	0.041
6	K	1.8	0.2	0.934	0.087
43. 2FCJ A	33N-G	11E-G			
33	N	0.7	0.8	0.000	1.582
34	G	0.1	0.8	0.000	0.379
11	E	2.7	0.6	0.000	9.059
12	G	0.2	0.7	0.251	0.902
44. 2G1P A	48S-R	29E-C			
48	S	2.3	0.3	0.565	0.275
49	R	2.6	0.3	0.565	0.408
29	E	0.1	0.6	0.000	0.000
30	C	1.7	0.1	0.000	0.000
45. 2G3W A	100R-E	79D-L			
100	R	0.4	1.0	0.000	0.000
101	E	0.2	0.1	0.000	0.000
79	D	0.2	5.6	0.227	0.000
80	L	0.6	5.6	0.227	0.000
46. 2G40 A	204R-R	148D-F			
204	R	0.3	0.4	0.000	0.000
205	R	0.7	0.3	0.000	0.000
148	D	0.2	1.2	0.000	0.000
149	F	5.0	1.2	0.000	11.062
47. 2G8S A	192G-I	161G-K			
192	G	1.1	0.3	0.000	11.312
193	I	0.8	0.2	0.000	10.461
161	G	0.0	0.4	2.408	0.000
162	K	0.5	0.4	0.935	0.000
48. 2GJL A	15E-H	39G-G			
15	E	4.7	0.0	0.000	0.000
16	H	4.7	0.0	0.000	0.000
39	G	0.0	1.0	0.374	0.000
40	G	0.1	0.9	1.325	0.000
49. 2GNP A	75E-S	299N-H			
75	E	1.2	0.2	0.000	0.000
76	S	1.2	0.1	0.000	0.000
299	N	1.8	0.1	0.149	0.000
300	H	2.0	0.1	0.000	0.000
50. 2HLJ A	66L-H	104L-A			
66	L	0.5	0.0	0.108	0.000
67	H	1.5	0.0	0.000	0.000
104	L	1.7	0.0	1.057	0.000
105	A	1.3	0.1	1.057	0.000
51. 2I4L A	400P-W	347R-V			
400	P	0.0	0.0	0.000	0.000
401	W	2.3	0.0	0.000	0.000
347	R	5.6	0.0	0.000	0.000
348	V	14.6	0.0	0.000	0.928
52. 2INU A	125F-G	182D-F			

125	F	0.2	1.2	0.816	13.612
126	G	0.1	1.0	0.000	1.216
182	D	1.3	0.2	0.000	2.189
183	F	2.9	0.2	0.000	2.189
53. 2INU A	246G-N	224E-H			
246	G	0.1	0.6	0.000	0.000
247	N	0.7	0.5	0.000	0.000
224	E	1.1	0.1	0.000	0.632
225	H	0.7	0.1	0.000	0.796
54.2IXD A	99K-L	5H-I			
99	K	0.4	0.1	0.000	0.000
100	L	1.7	0.1	0.000	0.150
5	H	8.0	0.1	0.254	0.409
6	I	8.7	0.0	0.254	6.325
55. 2NTP A	114T-I	156A-L			
114	T	31.6	0.0	0.251	3.315
115	I	23.6	0.0	0.251	3.315
156	A	3.8	0.0	0.825	69.038
157	L	4.1	0.0	0.825	69.038
56. 2NTP A	156A-L	179T-L			
156	A	3.8	0.0	0.825	69.038
157	L	4.1	0.0	0.825	69.038
179	T	1.3	0.2	0.000	0.781
180	L	1.8	0.1	0.000	1.386
57. 2NTP A	179T-L	200F-I			
179	T	1.3	0.2	0.000	0.781
180	L	1.8	0.1	0.000	1.386
200	F	2.8	0.1	0.000	0.000
201	I	1.9	0.0	0.000	0.000
58. 2NTP A	200F-I	231L-T			
200	F	2.8	0.1	0.000	0.000
201	I	1.9	0.0	0.000	0.000
231	L	2.2	0.0	0.000	0.313
232	T	2.3	0.0	0.000	0.118
59. 2NXV A	76R-Y	17M-F			
76	R	0.8	1.3	0.000	0.000
77	Y	2.9	0.2	0.000	81.573
17	M	1.9	0.0	1.699	51.394
18	F	6.0	0.0	0.945	55.892
60. 2O6S A	84G-N	108T-N			
84	G	0.0	1.0	0.110	0.000
85	N	0.0	1.1	0.000	0.000
108	T	1.3	0.8	0.360	0.000
109	N	1.1	0.5	0.360	0.000
61. 2O6S A	108T-N	132T-N			
108	T	1.3	0.8	0.360	0.000
109	N	1.1	0.5	0.360	0.000
132	T	0.9	0.6	0.516	0.129
133	N	0.7	0.6	0.516	0.000
62. 2O6S A	132T-N	156Q-N			
132	T	0.9	0.6	0.516	0.129
133	N	0.7	0.6	0.516	0.000

156	Q	2.1	0.2	1.043	0.000
157	N	1.8	0.2	0.511	0.000
63. 2O6S A	156Q-N	180D-N			
156	Q	2.1	0.2	1.043	0.000
157	N	1.8	0.2	0.511	0.000
180	D	0.2	0.1	0.000	0.000
181	N	0.1	0.4	0.159	0.000
64. 2OKT A	175S-L	198P-F			
175	S	2.2	1.4	0.336	0.000
176	L	2.3	1.4	0.336	0.000
198	P	0.0	0.0	0.000	0.000
199	F	0.4	0.5	0.000	0.000
65. 2OLO A	198G-K	6D-V			
198	G	0.1	0.5	0.443	0.000
199	K	1.8	0.5	0.242	0.000
6	D	9.5	0.3	0.000	0.000
7	V	14.6	0.3	0.000	31.177
66. 2OOK A	109G-E	85E-R			
109	G	1.1	0.2	0.000	6.295
110	E	6.3	0.2	0.000	4.226
85	E	2.9	0.1	3.160	0.411
86	R	3.0	0.1	3.160	0.411
67. 2OSV A	243F-T	224F-H			
243	F	12.9	0.2	0.000	0.000
244	T	20.7	0.0	0.000	0.000
224	F	5.4	0.1	0.000	59.714
225	H	3.8	0.1	0.000	2.472
68. 2OZT A	169S-W	196P-L			
169	S	4.4	1.0	0.197	0.000
170	W	4.5	0.5	0.314	0.000
196	P	0.0	0.0	0.000	0.000
197	L	0.0	0.0	0.000	0.000
69. 2QJJ A	214R-Y	238C-T			
214	R	3.9	0.0	0.000	0.000
215	Y	3.9	0.0	0.000	0.000
238	C	1.1	0.4	0.000	0.000
239	T	1.1	0.0	1.293	0.000
70. 2RKQ A	36P-R	105G-S			
36	P	0.0	0.2	0.127	0.000
37	R	0.3	0.2	0.127	0.000
105	G	0.0	3.4	0.000	0.096
106	S	0.1	1.2	0.000	1.363
71. 2UVJ A	183P-V	237V-M			
183	P	0.2	0.0	0.000	0.000
184	V	7.4	0.0	0.000	0.000
237	V	4.1	0.0	0.000	0.000
238	M	3.9	0.3	0.000	0.000
72. 2V0H A	364L-T	347F-V			
364	L	4.2	0.0	0.000	0.527
365	T	9.1	0.0	0.000	0.527
347	F	2.3	3.5	0.467	0.397
348	V	10.0	0.0	0.573	0.397

73. 2V3I A	281T-L	273F-Y			
281	T	12.7	0.1	0.000	0.000
282	L	5.7	0.1	0.000	0.000
273	F	3.4	0.2	0.000	0.000
274	Y	2.0	0.4	0.000	0.000
74. 2VUW A	759K-T	620S-S			
759	K	13.6	0.1	0.323	0.000
760	T	3.0	0.2	0.000	0.000
620	S	0.8	0.2	1.471	0.000
621	S	0.5	0.2	3.700	0.000
75. 2W7Z A	36Q-I	56H-L			
36	Q	2.8	0.1	0.000	0.000
37	I	6.7	0.1	0.000	0.000
56	H	1.2	0.3	0.260	0.000
57	L	2.3	0.3	0.260	0.000
76. 2W7Z A	71N-V	91R-V			
71	N	3.0	0.8	0.000	0.000
72	V	5.1	0.3	0.000	0.000
91	R	4.1	0.0	0.000	0.000
92	V	10.7	0.0	0.000	0.000
77. 2W7Z A	91R-V	111D-C			
91	R	4.1	0.0	0.000	0.000
92	V	10.7	0.0	0.000	0.000
114	D	0.3	0.4	0.227	0.000
115	C	0.7	0.4	0.227	0.000
78. 2W7Z A	111D-C	131L-V			
111	D	0.3	0.4	0.227	0.000
112	C	0.7	0.4	0.227	0.000
131	L	2.8	0.2	0.106	0.330
132	V	5.3	0.0	0.106	0.330
79. 2WHL A	265L-S	253W-K			
265	L	1.1	0.9	0.000	0.000
266	S	0.2	2.9	0.803	0.000
253	W	4.2	0.2	0.000	1.219
254	K	1.1	1.0	0.000	0.000
80. 2WNH A	283R-W	90S-I			
283	R	5.1	0.0	1.364	0.000
284	W	2.5	0.0	1.364	79.289
90	S	0.3	0.1	0.841	0.000
91	I	1.8	0.1	0.841	0.000
81. 2X5O A	206K-V	172V-A			
206	K	1.2	0.0	0.000	0.000
207	V	3.2	0.0	0.000	5.181
172	V	1.8	0.0	0.393	39.991
173	A	1.6	0.0	0.393	41.280
82. 2XHG A	410A-L	364E-V			
410	A	5.5	0.0	0.391	6.578
411	L	3.9	0.0	0.391	6.768
364	E	4.4	0.1	0.000	0.000
365	V	12.3	0.1	0.000	2.194
83. 2XT2 A	53E-C	73E-V			
53	E	0.8	0.1	0.313	0.000

54	C	1.7	0.1	0.185	0.000
73	E	4.3	0.0	0.159	0.787
74	V	5.7	0.0	0.159	1.098
84. 2Y24 A	297L-L	291I-R			
297	L	0.4	0.2	0.152	0.938
298	L	0.2	0.3	0.152	0.938
291	I	3.9	0.0	1.292	73.341
292	R	3.7	0.1	1.292	0.000
85. 2Z0D A	57N-F	67T-S			
57	N	0.6	0.2	0.822	0.000
58	F	0.2	0.4	0.822	0.000
67	T	0.1	0.9	0.000	0.000
68	S	0.1	0.9	0.000	0.000
96. 3DKR A	86A-K	15D-T			
86	A	2.2	0.0	1.254	0.000
87	K	1.9	0.0	1.254	0.000
15	D	0.4	0.7	0.000	0.000
16	T	0.4	0.4	0.000	5.305
87. 3DLQ R	190G-L	133R-S			
190	G	1.2	0.4	0.000	0.000
191	L	0.4	0.4	0.000	0.000
133	R	4.2	0.0	0.000	0.000
134	S	3.1	0.0	0.268	0.222
88. 3EU3 A	58P-V	92D-V			
58	P	0.0	0.0	0.000	0.000
59	V	17.6	0.0	0.000	0.000
92	D	1.2	1.6	0.137	1.285
93	V	4.1	0.4	0.137	1.285
89. 3EUR A	29E-Y	64K-L			
29	E	2.4	0.0	0.000	0.000
30	Y	7.0	0.0	0.000	59.955
64	K	1.0	0.1	3.524	0.000
65	L	2.6	0.1	2.491	0.000
90. 3F4S A	40P-I	74K-M			
40	P	0.0	0.0	0.000	2.395
41	I	3.2	0.0	0.000	2.395
74	K	0.4	0.7	2.084	1.328
75	M	0.9	0.2	2.084	22.428
91. 3FF1 A	327N-H	381P-Q			
327	N	3.4	0.0	0.138	1.075
328	H	1.5	0.3	0.000	0.000
381	P	0.2	0.0	0.000	0.000
382	Q	0.6	0.0	0.000	0.000
92. 3FRH A	125A-S	105R-R			
125	A	2.0	0.0	0.000	5.528
126	S	2.2	0.0	0.000	5.528
105	R	0.8	0.2	1.200	0.000
106	R	2.3	0.2	1.200	0.000
93. 3G5T A	62E-Q	38K-L			
62	E	1.0	0.1	0.000	0.000
63	Q	1.5	0.1	0.000	0.000
38	K	1.3	0.1	2.828	2.872

39	L	1.3	0.1	2.828	2.872
94. 3H2Z A	62G-V	44H-S			
62	G	0.7	0.2	0.000	0.000
63	V	0.8	0.1	0.000	0.000
44	H	0.6	0.2	0.907	0.000
45	S	3.1	0.1	0.111	0.000
95. 3H74 A	12A-V	41S-T			
12	A	1.0	0.8	0.176	0.000
13	V	0.4	1.2	0.176	0.000
41	S	1.1	0.1	0.000	0.000
42	T	3.5	0.1	0.000	0.000
96. 3HBN A	227N-K	159D-F			
227	N	0.0	1.8	0.380	0.000
228	K	0.5	0.2	0.199	0.000
159	D	3.7	0.1	0.959	0.118
160	F	4.1	0.1	0.709	14.789
97. 3ILX A	148L-N	121A-Y			
148	L	10.1	0.0	0.000	2.735
149	N	3.3	0.1	0.144	2.735
121	A	5.2	0.0	0.199	0.372
122	Y	1.3	0.9	0.199	0.372
98. 3IPF A	30G-V	49G-Q			
30	G	0.1	0.1	0.389	2.012
31	V	2.7	0.1	0.000	2.012
49	G	0.1	0.4	0.319	0.000
50	Q	1.5	0.4	0.319	0.000
99. 3JRN A	64R-F	10D-V			
64	R	1.1	0.1	0.521	0.317
65	F	1.2	0.1	0.355	92.657
10	D	6.0	0.0	0.320	0.483
11	V	8.4	0.0	0.320	76.333
100. 3K6M A	200E-T	167D-F			
200	E	0.1	0.1	0.105	0.000
201	T	1.8	0.1	0.000	0.000
167	D	0.1	0.8	2.233	0.132
168	F	0.7	0.7	1.386	0.132
101. 3KS3 A	140G-L	206C-V			
140	G	0.1	2.5	0.298	7.936
141	L	0.4	0.2	0.298	95.040
206	C	1.5	0.0	0.094	55.306
207	V	7.2	0.0	0.094	92.376
102. 3LHI A	186G-H	128D-V			
186	G	0.2	0.4	0.000	0.000
187	H	1.7	0.4	0.000	0.157
128	D	0.1	0.6	0.343	0.000
129	V	3.0	0.6	0.343	16.201
103. 3MD7 A	235K-R	204D-V			
253	K	0.2	0.4	0.311	0.000
254	R	0.5	0.4	0.311	0.000
204	D	1.9	0.4	0.108	0.000
205	V	3.2	0.5	0.108	0.000
104. 3MDW A	35D-A	18N-V			

35	D	0.0	5.5	0.000	0.000
36	A	0.0	5.5	0.000	0.000
18	N	3.3	0.1	0.962	0.000
19	V	5.3	0.1	0.318	0.000

105. 3MQD A 380N-V 353N-F

380	N	1.2	0.2	0.000	0.000
381	V	4.9	0.1	0.000	0.000
353	N	1.0	0.3	0.000	0.000
354	F	2.4	0.1	0.000	0.000

106. 3N1M C 779H-L 727T-T

779	H	1.9	0.2	0.000	0.000
780	L	1.4	0.0	0.000	0.000
727	T	1.0	0.1	17.546	4.948
728	T	3.3	0.1	18.283	4.948

107. 3N6Z A 206G-V 170G-Y

206	G	0.8	0.2	0.000	1.284
207	V	0.5	0.8	0.000	1.590
170	G	2.6	0.1	0.282	37.881
171	Y	0.4	0.2	0.282	37.596

108. 3N6Z A 243G-R 206G-V

243	G	1.2	0.4	0.128	0.000
244	R	0.6	0.4	0.128	0.000
206	G	1.2	0.2	0.165	7.783
207	V	1.0	0.2	0.165	7.783

109. 3N6Z A 273G-Q 243G-R

273	G	1.2	0.0	0.114	0.557
274	Q	0.9	0.0	0.114	0.557
243	G	1.2	0.4	0.128	0.000
244	R	0.6	0.4	0.128	0.000

110. 3N6Z A 285N-S 316N-C

285	N	0.4	0.2	0.469	0.000
286	S	0.2	0.2	0.283	0.000
298	N	3.0	0.4	0.000	0.000
299	C	7.8	0.4	0.000	0.000

111. 3NRE A 205G-W 230P-C

205	G	0.5	0.7	0.000	0.000
206	W	0.2	3.3	0.000	0.000
230	P	0.0	0.1	0.000	0.332
231	C	1.0	0.1	0.000	26.575

112. 3OQI A 60Q-S 30R-H

60	Q	2.5	0.2	0.306	8.850
61	S	11.2	0.3	0.650	13.218
30	R	1.4	0.1	1.700	0.000
31	H	2.6	0.1	0.525	0.000

113. 3PB6 X 160A-R 214P-V

160	A	0.3	0.1	6.888	0.000
161	R	0.7	0.1	6.888	0.000
214	P	0.0	0.0	0.000	0.541
215	V	7.1	0.0	0.000	45.917

114. 3PG6 A 710T-S 719Y-G

710	T	1.0	0.3	0.000	0.000

711	S	0.9	0.3	0.000	0.000
719	Y	0.7	0.2	0.000	0.000
720	G	0.5	0.2	0.000	0.000
115. 3PZS A	12V-F	41S-N			
13	V	6.6	0.0	0.000	0.000
14	F	4.6	0.2	0.000	0.000
42	S	2.4	0.3	0.000	0.000
43	N	0.4	0.3	0.000	0.000
116. 3QUF A	269G-V	186A-F			
269	G	0.1	0.1	0.268	0.000
270	V	2.2	0.0	0.268	0.000
186	A	1.2	0.1	0.000	0.000
187	F	1.7	0.0	0.000	0.000
117. 3R9P A	363P-I	339G-I			
363	P	0.0	0.0	0.722	0.325
364	I	16.5	0.0	0.722	80.552
339	G	0.2	0.1	0.000	0.291
340	I	8.5	0.0	0.000	0.291
118. 3UR8 A	324L-F	307L-F			
324	L	0.8	0.3	0.000	0.000
325	F	0.9	0.1	0.000	0.000
307	L	4.1	0.0	0.210	21.025
308	F	1.7	0.0	0.210	21.025
119. 3VII A	462I-Y	446L-M			
462	I	1.1	0.3	0.000	31.541
463	Y	1.9	0.3	0.000	31.541
446	L	2.3	0.2	0.000	7.511
447	M	0.7	0.6	0.000	4.120
120. 3VMV A	203N-L	225D-I			
203	N	0.8	1.1	0.000	0.000
204	L	0.7	1.1	0.000	0.000
225	D	1.5	1.3	0.000	0.000
226	I	1.2	1.3	0.000	0.000
121. 3VMV A	225D-I	248N-V			
225	D	1.5	1.3	0.000	0.000
226	I	1.2	1.3	0.000	0.000
248	N	0.4	0.8	0.000	0.000
249	V	0.2	1.9	0.000	0.000
122. 3VNY A	244A-E	290S-C			
244	A	2.0	0.2	0.000	0.000
245	E	0.3	0.2	0.000	0.000
290	S	3.7	0.1	0.000	0.000
291	C	9.3	0.1	0.000	0.000
123. 4A35 A	258R-W	282P-T			
258	R	3.4	0.1	0.000	0.000
259	W	6.3	0.1	0.000	0.000
282	P	0.0	0.1	0.000	0.000
283	T	0.1	0.1	0.000	0.000
124. 4AQ4 A	162K-C	240D-C			
162	K	2.5	0.3	0.000	0.000
163	C	2.6	0.3	0.000	0.000
244	D	0.1	2.2	0.000	0.000

245	C	0.7	0.8	0.000	0.000
125. 4BA1 A	243K-G	222D-A			
243	K	0.0	1.9	0.000	0.000
244	G	0.0	1.6	0.000	0.000
222	D	0.8	0.1	1.699	0.000
223	A	0.5	0.1	1.699	0.000
126. 4DWD A	201G-Y	225P-V			
201	G	0.1	0.9	0.121	0.000
202	Y	4.8	0.7	0.121	0.000
225	P	0.0	0.0	0.000	0.000
226	V	1.7	0.1	0.000	0.000
127. 4EQB A	230A-I	168S-I			
230	A	3.4	0.0	0.000	46.614
231	I	3.1	0.0	0.000	50.019
168	S	0.6	0.2	0.701	20.539
169	I	3.8	0.0	0.166	20.539
128. 4EXK A	223A-M	146A-L			
223	A	0.6	0.1	0.265	0.000
224	M	5.0	0.0	0.265	0.000
146	A	0.7	0.2	0.000	4.277
147	L	1.0	0.0	0.000	4.721
129. 4GFI A	179G-W	203P-L			
179	G	0.1	0.6	0.000	0.000
180	W	0.2	0.6	0.000	0.000
203	P	0.0	0.0	0.000	0.000
204	L	0.0	0.2	0.000	0.000

[a] TANGO could not be run on 20 proteins with long chains (which contained 41 motifs in total). The files are: 1X38A, 2CHOA, 2CXNA, 2D5WA, 2EP1XA, 2GJ4A, 2NOOA, 2VBKA, 2VFOA, 2WMKA, 2X6WA, 2XHGA, 2Y53A, 2YEQA, 3FO3A, 3M8UA, 3O83A, 3QPAA, 3SUVA and 4EU9A.
